# Processing and Structure of the Lantibiotic Peptide Nso From the Human Gut Bacterium *Blautia obeum* A2-162 analysed by Mass Spectrometry

**DOI:** 10.1038/s41598-018-28248-6

**Published:** 2018-07-04

**Authors:** Cristina Gherghisan-Filip, Gerhard Saalbach, Diane Hatziioanou, Arjan Narbad, Melinda J. Mayer

**Affiliations:** 1 Quadram Institute Bioscience, Gut Microbes and Health Institute Strategic Programme, Norwich Research Park, Norwich, NR4 7UA UK; 2 John Innes Centre, Department of Biological Chemistry, Norwich Research Park, Norwich, NR4 7UH UK; 3 Present Address: University of East Anglia, UEA, Norwich Medical School, Norwich, NR4 7TJ UK

## Abstract

A previously reported gene cluster encoding four nisin-like peptides, three with the same sequence (NsoA1-3) and the unique NsoA4, produced antimicrobial activity in the presence of trypsin after heterologous expression in *Lactococcus lactis*. Protein extracts were separated by SDS gel electrophoresis or immunoprecipitation using an antibody to the NsoA2 leader. Tryptic peptides observed by LC-MS/MS covered the complete sequence of preNsoA1-3 and part of the leader sequence of preNsoA4 and confirmed the expression and the predicted sequences of the preNsoA peptides. Further, the data revealed that the preNsoA1-3 peptides were partly modified with dehydrations and formation of lanthionine rings. A certain amount of fully modified preNsoA1-3 was observed. Details of modifications of the core peptide and the C-terminal tryptic peptide TATCGCHITGK covering rings D and E indicated that 22% of these preNsoA1-3 peptides were completely modified. A lower amount of ring formation is estimated for rings A-C. Intact masses of immunoprecipitation-derived peptides determined by LC-MS accurately matched the expected preNsoA precursor peptides. The most abundant peptides detected were preNsoA2-3-8H_2_O followed by preNsoA1-8H_2_O and other states of dehydration. The results confirm incomplete processing of preNsoA peptides in the heterologous system, with the formation of a certain amount of fully modified peptides.

## Introduction

Lantibiotics are small, ribosomally-synthesized, post translationally modified lanthipeptides with antimicrobial activity^1^. They are both produced by and active against Gram-positive bacteria, including important antibiotic resistant pathogens^2,3^. Their stability, specificity and ability to act at nanomolar concentrations makes them promising candidates for the development of novel antimicrobials – both to target specific microbes without disrupting the complex beneficial microbiota of gut or food microbiomes, and to provide new tools to combat multidrug resistant bacteria^2^. They already have application in food preservation and are being investigated for efficacy in a range of therapeutic applications^4–7^.

Nisin A is one of the best characterized examples of a lanthipeptide. Enzyme-mediated modifications are responsible for converting the ribosomally synthesized, immature and inactive prepeptides into peptides that contain the characteristic features of lantibiotics^8^. The maturation of lantibiotics takes place via two main reactions. Serine and threonine residues in the core region are dehydrated to dehydroalanine and dehydrobutyrine (Dha and Dhb) then cysteine thiols are stereo selectively added to the dehydroamino acids by Michael addition to form thio-ether cross-links, called lanthionine and methyl-lanthionine rings respectively^8–10^. Due to these unusual amino acids, complete and correctly processed nisin can exhibit antimicrobial activity and various mechanisms of action^11^.

The genes involved in lantibiotic biosynthesis, regulation and immunity are usually clustered in an operon. Nisin gene clusters typically encode modification enzymes NisB, which performs the serine and threonine dehydration on the prepeptide encoded by *nisA*, and NisC, a zinc-dependent metalloprotein which cyclises dehydrated residues to cysteines^9^. After modification, the leader peptide is cleaved by the serine protease NisP following export by ABC-transporter NisT^10^. Mature nisin A contains three dehydrated amino acids and five thioether bridges, and the same pattern has either been demonstrated or predicted in natural nisin variants Z, F, Q, U, U2, P and H^12–18^. The two component sensor-histidine kinase system NisRK regulates expression and coordinates autoinduction of the nisin A promoter, P_nisA_, by the mature nisin product^8^. The products of the remaining genes *nisFEG* and *nisI* mediate immunity, although a *nisI* homologue is absent from the nisin H cluster^14^. Nisin has been expressed heterologously in *L. lactis* and *Enterococcus* sp^19^, and a fully modified nisin precursor with antimicrobial activity after trypsin treatment was synthesized *in vitro* using a rapid translation system containing genes *nisA, nisB* and *nisC*^20^. The nisin biosynthetic machinery has also been shown to be capable of modifying other non-nisin peptides^21–23^ and a recent publication demonstrated its value in enabling novel lantibiotic production^24^.

The defining structural elements of lantibiotics offer not only structural and biological function to these peptides, but they also make lantibiotics resistant to proteolysis from internal proteases and tolerant to oxidation^25^. On the other hand, some lantibiotic peptides possess limitations for routine and widespread use, such as instability and insolubility at physiological pH and their predisposition to being degraded by intestinal proteases^11,26^.

To overcome these kinds of limitations and identify antimicrobials suitable for use in the gastrointestinal tract, searches have been performed for new lantibiotics from gut bacteria. Ruminococcin A, derived from a commensal bacterium of the human intestinal microbiota, *Ruminococcus gnavus* E1, exhibits trypsin-dependent anti-*Clostridium perfringens* activity^27^, and human intestinal isolate *Bifidobacterium longum* DJO10A encodes a lantibiotic cluster and antimicrobial activity against a related strain^28^. *In silico* screening of human gut microbiota genomes sequenced in the metaHIT project identified extensive carriage of bacteriocin genes^29^, and O’Connor *et al*.^14^ described the first nisin variant to be produced from a gut bacterium - nisin H - from a porcine gut. The identification and characterisation of a gene cluster from the human gut isolate *Blautia obeum* A2-162 encoding novel nisin-like peptides NsoA1-3 and NsoA4 has previously been reported^30^. The cluster lacks a processing protease gene, and antimicrobial activity of the host strain could only be detected in the presence of trypsin. Since molecular tools to manipulate the original *B. obeum* producer are not available, the entire *nso* cluster was cloned in *L. lactis*, where it was possible to obtain heterologous expression of the Nso peptides. Antimicrobial activity of the Nso peptides was observed after treatment with trypsin, presumably releasing the biologically active Nso core peptide from the leader sequence. The trypsin-treated Nso peptides were highly active against two clinically important pathogens, *C. perfringens* and *Clostridium difficile*^30^. In the present work, we aim for the characterization of the processing of the NsoA peptides in *L. lactis* expressing the *nso* operon, leading to the post-translational modifications essential for activity. Nso peptides were prepared by immunoprecipitation as well as from SDS gel bands and analysed by LC-MS/MS to characterise the specific steps of the formation of the active Nso peptides. The results show that the Nso peptides are incompletely processed by the nisin O processing machinery in *L. lactis*, but confirm that dehydration and ring formation do occur leading to the formation of fully modified peptides.

## Results and Discussion

### Predicted structure and processing of Nso peptides

**Figure 1:**
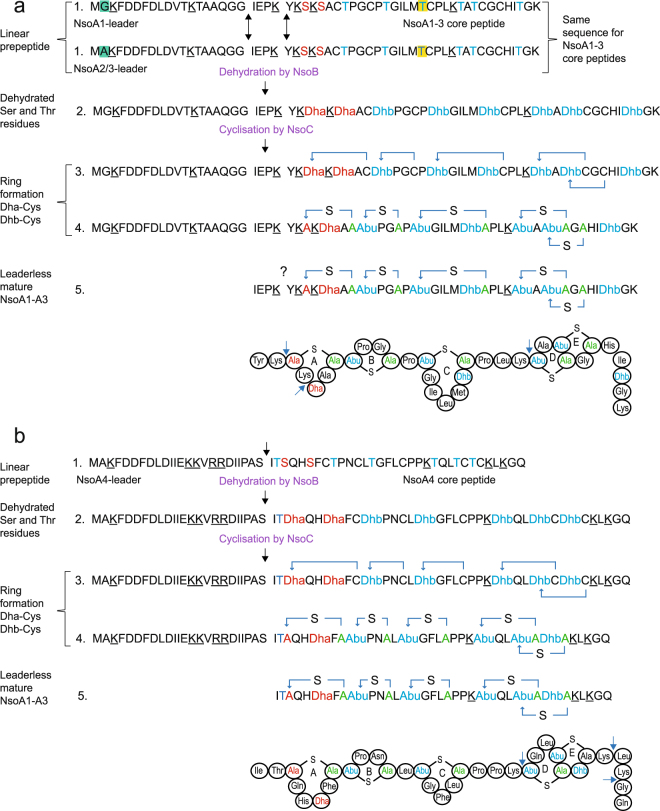
Predicted sequences and processing steps of the Nso peptides: (**a**) preNsoA1, (**b**) preNsoA4. Uniprot accession numbers: preNsoA1: A0A286N5V5, preNsoA2/3: A0A286N5V7, preNsoA4: A0A286N5V8. Black arrows show two putative leader cleavage sites for preNsoA1-3, which have the same sequence except for residue 1, and one for preNsoA4. The trypsin cleavage sites are underlined in the linear sequences and indicated with a blue arrow head in the ringed structures. The proposed steps of the processing of preNsoA peptides include (**1**) linear preNsoA precursor peptide with residues that will be dehydrated by NsoB in red (serine) and in blue (threonine); one threonine residue which is also present in nisin variants H, U, U2 and P but absent from A, Z, F and Q is highlighted in yellow. (**2**) Linear peptide with dehydrations catalysed by NsoB resulting in dehydroalanine (Dha) and dehydrobutyrine (Dhb). (**3**) (Me)Lan ring formation catalysed by NsoC between residues indicated by a blue arrow. (**4**) PreNsoA precursor peptide with dehydrations and rings formed. (**5**) Leaderless Nso core peptide with dehydrations and rings formed.

The *nso* gene cluster contains four predicted *nso* structural genes. The biosynthesis of the active Nso peptides as predicted in analogy to the sequence and processing of nisin and other lantibiotic peptides^31–33^ is illustrated in Fig. 1. Of the deduced peptide sequences, three (preNsoA1 to A3) are identical except for a single amino acid residue difference in the preNsoA1 leader peptide. The preNsoA4 sequence is unique for both the leader and the core peptide. The proposed processing steps include the expression of a linear preNsoA peptide and the introduction of modifications such as dehydration of serine and threonine residues and the subsequent ring formation between the dehydrated amino acids and the cysteine residues.

The *nso* operon does not encode a protease for propeptide cleavage. In the native producer *B. obeum* cleavage of the preNsoA peptides might be performed by unspecific proteases encoded within its genome, as is seen with lantibiotics subtilin and mutacin^34,35^. Potential sites suggested for the cleavage based on comparison with nisin and other lantibiotics^3,33^ are indicated in Fig. 1 (black arrows). The site 22P23K↓24Y in preNsoA1-3 resembles the C-terminal PQ or PR motif frequently observed in lantibiotic leader sequences^33^, and would generate a core peptide of similar length and similar N-terminal structure to nisin A. In both cases, it also represents a cleavage site for trypsin. In addition, the potential leader sequence of preNsoA1-3 contains a double-glycine motif (GG) known as a protease cleavage site in some type II and a few type I lantibiotics^3,36^. In preNsoA4 a clear PQ/PR or GG motif is not present. The site 24 S↓25I is suggested as potential cleavage site since it generates a core peptide with an N-terminus similar to that of nisin (ITS).

### Analysis of immunoprecipitated preNsoA1-3

**Figure 2:**
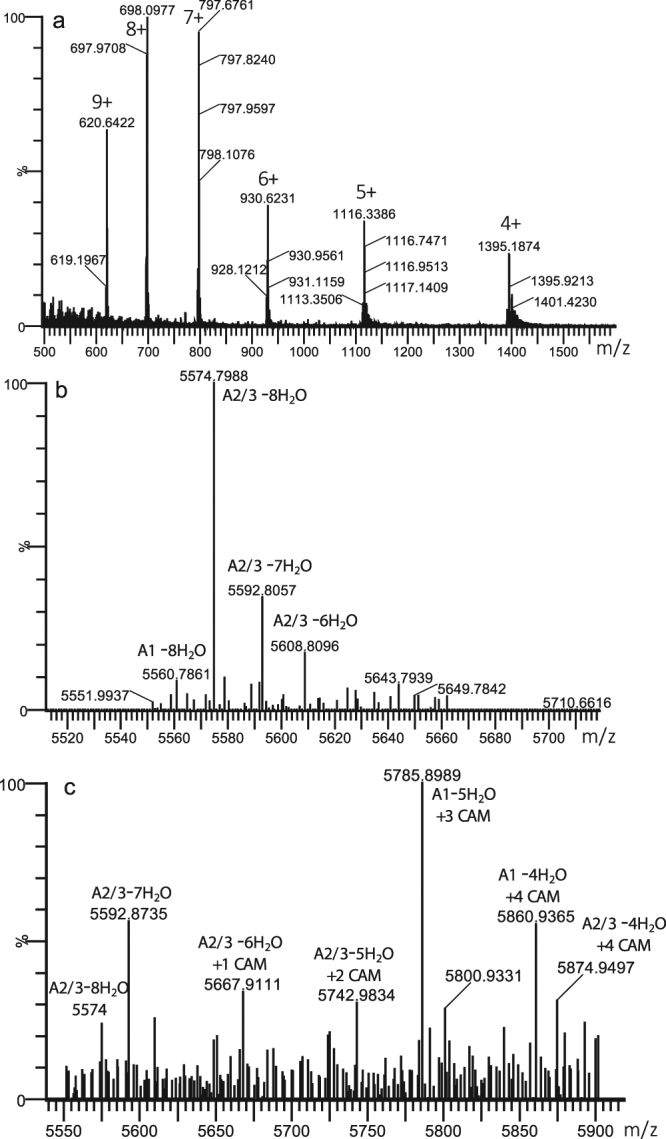
Spectrum from LC-MS analysis of immunoprecipitated preNsoA1-3 peptide. (**a**) raw spectrum showing a series of charge states from 4+ to 9+. (**b**) deconvoluted spectrum from (**a**) using the MaxEnt3 tool in the Masslynx software (Waters) showing 2 abundant peaks at *m/z* 5574.72 (1+) and *m/z* 5560.71 (1+) matching the expected masses of preNsoA2-3 (*m/z* 5574.70, 1+) and preNsoA1 (*m/z* 5560.68, 1+). Lock mass correction is not possible with the MaxEnt3 tool. For a more accurate calculation see text and Supplementary Information: Fig. S2. In addition, lower dehydration states are present. (**c**) MaxEnt3 deconvoluted spectrum of charge state 7+ of IP generated preNsoA1-3 treated with iodoacetamide to alkylate free cysteine residues. A portion of 7- and 8-fold dehydrated preNsoA2-3 with no CAM modification is present together with several different lower dehydration states combined with several CAM modifications. For more details see Supplementary Information: Fig. S2.

For the confirmation of the predictions made in Fig. 1, experiments were carried out to prepare and analyse preNsoA/NsoA peptides. The antibody against the NsoA2-3 leader peptide was used for the preparation of preNsoA1-3 peptides using immunoprecipitation from UKLc10 cell cultures expressing the *nso* operon, with the aim of studying the structure and potentially the activity of Nso peptides. LC-MS was used to analyse modifications and the spectra of the eluted samples showed a predominant series of charge states (Fig. 2). The isotope patterns of this predominant series match the expected pattern for preNsoA2-3 (Supplementary Information: Fig. S1). Monoisotopic masses (*m/z*) were obtained after lock mass correction and used to calculate the measured mass (Supplementary Information: Fig. S2A). In this way, for the most abundant isotope pattern a value of *m/z* 5574.6889 (1+) matching the predicted mass of the 8-fold dehydrated preNsoA2-A3 peptide (*m/z* 5574.696, 1+) with an error of 1.3 ppm was obtained. A similar but much less abundant series of charge states was observed for *m/z* 5560.6791 (1+) fitting to the 8-fold dehydrated preNsoA1 peptide (*m/z* 5560.6803, 1+) with an error of 0.22 ppm. In addition, in different LC-MS runs several weak peaks indicating incomplete dehydrations of both preNsoA1 and preNsoA2-3 were also observed (Supplementary Information: Figs S3 and S4). LC-MS/MS analysis of a trypsin digest of the IP samples confirmed the presence of tryptic peptides from preNsoA1-3 (Supplementary Information: Scaffold file 1). The analysis of tryptic peptides for the localisation of modifications was further extended by applying trypsin digestion to gel slices containing preNsoA peptides (see following chapter).

To test the immunoprecipitated preNsoA1-3 peptides for the presence of rings, an aliquot was treated with iodoacetamide to alkylate free cysteine residues, and analysed by LC-MS. Interestingly, a portion of preNsoA2-3 with 8 or 7 dehydrations and no carboxymethylation (CAM) modification was observed indicating the presence of all rings (Fig. 2c, Tables 1, 2 and Supplementary Information: Fig. S4). In addition, a series of different dehydration states with several CAM modifications of preNsoA2-3 was present showing an inverse correlation between number of dehydrations and number of CAM modifications (Tables 1 and 2).

**Table 1:** Observed masses and assigned dehydration and cysteine modification states for IAA-treated preNsoA1-3 peptides.

Observed *m/z*	Deconvoluted Da	Observed *m/z*	Deconvoluted Da	Observed *m/z*	Deconvoluted Da
797.27	5573.84	821.29	5741.98	840.14	5873.93
799.84	5591.83	823.84	5759.83	842.70	5891.85
802.4	5609.75	827.43	5784.96	845.27	5909.84
807.98	5648.81	829.3	5798.05	848.80	5934.55
810.56	5666.87	831.87	5816.04	853.30	5966.05
813.14	5684.93	834.57	5834.94	856.00	5984.95
818.85	5724.90	838.14	5859.93	858.44	6002.03

Shown are masses of observed monoisotopic peaks selected from 7+ charged isotope patterns from acquired raw spectra and their corresponding deconvoluted masses.

**Table 2:** Observed masses and assigned dehydration and cysteine modification states for IAA-treated preNsoA1-3 peptides. Shown are calculated theoretical masses of preNsoA1 and preNsoA2-3 peptides with different numbers of dehydrations combined with different numbers of CAM modifications of cysteine residues.

#CAM on Cys	0	1	2	3	4	5	6	7	8
**preNsoA1**	**Number of dehydrations**								
0	5703.76	5685.75	5667.74	5649.73	5631.72	5613.71	5595.70	5577.69	5559.68
1	5760.78	5742.77	**5724.76**	5706.75	5688.74	5670.73	5652.72	5634.71	5616.70
2	5817.80	5799.79	5781.78	5763.77	5745.76	5727.75	5709.74	5691.73	5673.72
3	5874.82	5856.81	5838.80	5820.79	5802.78	**5784.77**	5766.76	5748.75	5730.74
4	5931.84	5913.83	5895.82	5877.81	**5859.80**	5841.79	5823.78	5805.77	5787.76
5	5988.86	5970.85	5952.84	**5934.83**	5916.82	5898.81	5880.80	5862.79	5844.78
**preNsoA2-3**	**Number of dehydrations**								
0	5717.77	5699.76	5681.75	5663.74	5645.73	5627.72	**5609.71**	**5591.70**	*5573.69*
1	5774.79	5756.78	5738.77	5720.76	5702.75	**5684.74**	**5666.73**	**5648.72**	5630.71
2	5831.81	5813.80	5795.79	5777.78	**5759.77**	**5741.76**	**5723.75**	5705.74	5687.73
3	5888.83	5870.82	5852.81	**5834.80**	**5816.79**	**5798.78**	5780.77	5762.76	5744.75
4	5945.85	5927.84	**5909.83**	**5891.82**	**5873.81**	5855.80	5837.79	5819.78	5801.77
5	**6002.87**	**5984.86**	5966.85	5948.84	5930.83	5912.82	5894.81	5876.80	5858.79

Bold numbers have been observed in the LC-MS analysis given in Table 1 above. A portion of fully (7- or 8-fold) dehydrated preNosA2-3 with no CAM (underlined italics) is present indicating complete modification including formation of all 5 rings. A series of incomplete dehydrations with increasing number of CAM of preNosA2/3 (all bold) is also present indicating dehydration dependent ring formation.

Without IAA treatment, the specific peptide preNsoA1 with 8 dehydrations has always been observed at a lower abundance than preNsoA2-3 (Fig. 2b), and lower dehydration states were probably too weak to be detected and would also overlap with the stronger preNsoA2-3 versions. Surprisingly, after IAA treatment the most abundant peak could specifically be assigned to preNsoA1-5H_2_O + 3CAM with an observed versus theoretical *m/z* of 5785.7417 and 5785.7764 (6 ppm error) (as calculated according to Supplementary Information: Fig. S4, see also Fig. 2c). Only three other versions of modified preNsoA1 were found compared to a total of 16 for preNsoA2-3 (Tables 1 and 2). The observed high abundance of preNsoA1-5H_2_O + 3CAM might be due to the specific behaviour of this version on the LC-MS system. The results from the IAA treatment show that dehydration is partially incomplete, subsequently leading to failure of the dehydration dependent ring formation, while all rings are formed in a portion of the peptides which are 7- to 8-fold dehydrated.

The preNsoA4 peptide could not be isolated by immunoprecipitation since the preNsoA4 leader sequence is significantly different from the preNsoA2-3 leader sequence against which the antibody used had been raised. Based on the results from the analysis of gel slices (see following chapter), preNsoA4 seems to be expressed at a much lower level than preNsoA1-3 (low spectrum count, Table 3). The preNsoA4 peptide has tryptic cleavage sites within the predicted leader sequence but none in the region of the predicted leader cleavage sites (see Fig. 1). The C-terminal peptide comprising rings D and E could be generated by trypsin in a similar manner to preNsoA1, but only 3 PSMs were observed and it is truncated by 4 amino acid residues by a trypsin cleavage after ring E. Taken together, preNsoA4 does not seem to play a role in the antimicrobial activity observed previously in the presence of trypsin^30^.

**Table 3:** List of PSMs for tryptic peptides observed by LC-MS/MS analysis of the 6 kDa gel slices with *m/z* (1+) for unmodified versions. MC = missed cleavage.

nso peptide	domain	tryptic peptide	*m/z* (1+)	MC	PSMs
A4	N-term leader	FDDFDLDIIEK	1369.6521	0	11
A4	N-term leader	FDDFDLDIIEKK	1497.7471	1	8
A4	N-term leader	AKFDDFDLDIIEK	1568.7842	1	30
A4	N-term leader	AKFDDFDLDIIEKK	1696.8792	2	26
A4	C-term core	TQLTCTCK	897.4168	0	3
A1-3	N-term leader	FDDFDLDVTK	1214.5575	0	36
A1	N-term leader	GKFDDFDLDVTK	1399.6739	1	65
A2-3	N-term leader	AKFDDFDLDVTK	1413.6896	1	129
A2-3	full leader	AKFDDFDLDVTKTAAQGGIEPK	2366.1874	2	7
A1-3	C-term leader	TAAQGGIEPK	971.5156	0	31
A1-3	C-term leader	TAAQGGIEPKYK	1262.6739	1	8
A1-3	N-term core	SACTPGCPTGILMTCPLK	1792.8464	0	166
A1-3	N-term core	SKSACTPGCPTGILMTCPLK	2007.9734	1	6
A1-3	N-term core	YKSKSACTPGCPTGILMTCPLK	2299.1317	2	4
A1-3	full core	SKSACTPGCPTGILMTCPLKTATCGCHITGK	3080.4528	2	1
A1-3	C-term core	TATCGCHITGK	1091.4972	0	123

### LC-MS/MS analysis of tryptic peptides from preNsoA peptides

As described previously, a polyclonal anti-leader peptide antibody raised from synthesized N-terminal acetylated NsoA2-3 leader peptide detected a band on Western blots around 6 kDa corresponding to the expected size of the Nso precursor peptides^30^ (Supplementary Information: Fig. S5). The bands were well defined without additional signals around them. In particular, no band or smear below 6 kDa was visible indicating that possibly modified peptides did not migrate separately and were not missed when cutting out the bands. No bands were observed which could represent the cleaved leader peptide (~2 kDa). On Coomassie stained gels, no clear differences in protein band patterns between strains expressing or not expressing the preNso peptides were observed.

Eight bands from the 6 kDa region of stained SDS-gels separating cell extracts (CE) and TCA-precipitated supernatants (TCA) from UKLc10 cultures expressing p*nso* (both with and without pTG*nsoA3_nsoA4*) were cut out and treated with trypsin before LC-MS/MS analysis. No significant differences could be observed between those sample types and the following results are combined from all eight samples. Results for individual gel slices are provided in Supplementary Information (Scaffold Files 2 and 3, where samples are labelled pnso and pnso +34 combined with CE and TCA).

Using standard parameters for the database search with 2 missed cleavages and at least 95% probability for peptide matches, a total of 654 peptide spectra matches (PSMs) (Table 3) were observed covering 100% of the preNsoA1-3 peptide sequences and 39% of the preNsoA4 sequence, confirming the sequences predicted from the *nso* gene cluster. This included 276 PSMs for the two tryptic peptides covering the complete predicted leader peptide of preNsoA1-3, and 299 PSMs for the 2 tryptic peptides covering the complete core peptide of preNsoA1-3. In addition, 75 PSMs were detected for the N-terminal tryptic peptide of the predicted leader peptide of preNsoA4, but only 3 PSMs for a tryptic peptide from the C-terminus of the core region of preNsoA4 were found (Table 3). The results confirmed the presence of preNso peptides in the 6 kDa gel area.

### Dehydration and ring formation

ESI-MS/MS has been proven a useful tool for analysing modifications and ring topology of nisin and other lantibiotic peptides^37–41^. The masses of the detected tryptic peptide are given in Table 3 as *m/z* (1+). The mass would be changed by −18 for each dehydration of S and T residues, and by +57 for each CAM modification of C residues. The ring formation itself does not change the mass.

Information regarding ring topology is based on the suppression of fragmentation within ring regions. The presented LC-MS/MS data show that all detected tryptic peptides from the core peptide region of preNsoA1-3 carried at least one of the modifications used in the database search, whereas all peptides derived from the leader sequences were unmodified (Supplementary Information: Scaffold file 2).

The peptides from the core peptide region were modified differently. All S and T residues (see Fig. 1) were detected unmodified and dehydrated in different combinations with every S or T residue dehydrated at least once. Dehydration was also observed on the T residue which is only present in some nisin variants^14^ (see Fig. 1). Cysteine residues were mostly modified with carbamido-methylation (CAM, C + 57), but groups of peptides with no CAM modification were also observed. The latter result can be considered as an indication of lanthionine ring formation since cysteine residues engaged in ring formation would not be available for CAM modification.

### Tryptic digests support the presence of a fully modified core peptide

**Figure 3:**
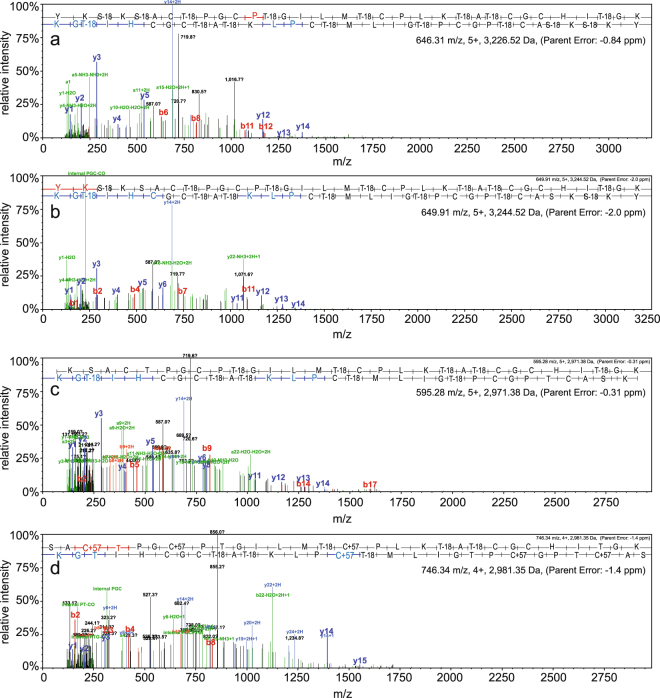
MS/MS spectra for NsoA1-3 core peptides generated by missed trypsin cleavages. All shown spectra were generated in the Scaffold software (see Supplementary Information: Scaffold file 3) and exported in the original format. b- and y-ions are shown in red and blue, respectively, and are assigned to the sequence on top of each spectrum. For fragment masses see Tables S4-7 (Supplementary Information). Core peptides starting with YKSK (**a**,**b**) and with SK (**c**) exhibit only few fragment peaks indicating poor fragmentation. In particular, fragments from ring C, D, E regions are missing, indicating that those rings are indeed present. Reproducible assignments have been made to y-ions from the linear region at the C-terminus and the short linear hinge region PLK located between rings C and D (see Fig. 1). Taken together, the spectra in (**a,b,c**) indicate that all five rings have been formed in the core peptide. (**d**) Spectrum for peptide starting SAC; the N-terminal trypsin cleavage producing this peptide has occurred inside the potential ring A region (see Fig. 1), excluding the presence of ring A. In addition, the total precursor mass indicates that three C residues are CAM modified and also some internal fragments (e.g. y14, y15) indicate that not all rings are present (for more details see Supplementary Information: Scaffold file 3).

Trypsin is the most common protease for generating peptides for MS analysis. The preNsoA peptides contain several trypsin cleavage sites, some of which would be in or directly adjacent to potential ring structures (see Fig. 1). It has been reported that in this case the sites can be protected against trypsin cleavage^42^. With preNsoA1-3 peptides this would essentially generate the predicted core peptide, which was not notably represented in the results shown in Table 3. The results in Table 3 clearly indicate that all trypsin cleavage sites can efficiently be cleaved. However, peptides as large as the core peptide and with ring structures can escape LC-MS detection. Therefore, another database search was performed with 3 missed cleavages and no filter for peptide probabilities (Supplementary Information: Scaffold file 3). In this way, several PSMs for the NsoA1-3 core peptide (with different tryptic N-termini) with mostly low or very low statistical probability caused by poor fragmentation were detected. Interestingly, they were predominantly found in one gel slice from a TCA precipitated sample, which could be caused by more complete processing but also by random variation of trypsin cleavage leaving more missed cleavages. Together with the high precursor mass accuracy (<2 ppm), the poor fragmentation and the occurrence of fragments from the linear C-terminal part and the hinge region (PLK) actually support the presence of rings (Fig. 3). The latter strongly suggests that those fully modified core peptides are indeed present, and the detected versions with missed trypsin cleavages and predicted modifications are listed in Tables S1–S3 (Supplementary Information). The PSMs for core peptides with an N-terminus starting before ring A (starting with YKSK or SK) mostly show a high degree of dehydration and no CAM modification on cysteine residues indicating the presence of all rings (A-E). The PSMs for core peptides starting with SAC cleaved by trypsin inside potential ring A indicate the presence of between 1–5 CAM modifications and 1–6 dehydrations, indicating incomplete modification and absence of some or all rings.

### Examination of the core peptide N-terminus

**Figure 4:**
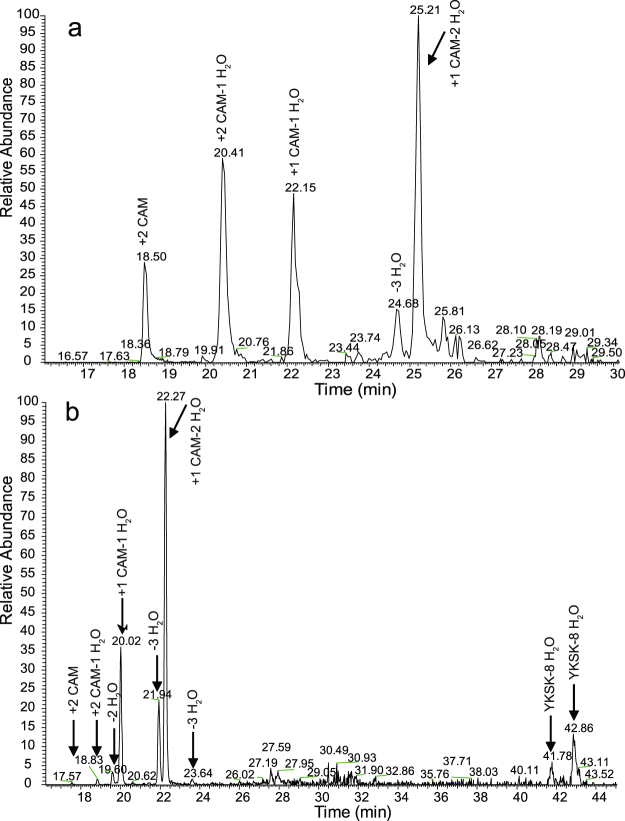
LC-MS elution profiles of differently modified version of the tryptic peptide TATCGCHITGK only (**a**) and the fully modified tryptic peptide YKSKSACTPGCPTGILMTCPLKTATCGCHITGK (YKSK) compared to TATCGCHITGK (**b**). (**a**) and (**b**) are extracted ion chromatograms from different LC-MS runs. In (**b**) the left group of peaks corresponds to the TATCGCHITGK peptides (similar to (**a**)). For comparison see Table S1 and Table 5. Interestingly, the fully modified tryptic peptide YKSK elutes at two specific elution times (41.78 min and 42.86 min) which might be related to the presence/absence of rings.

The abundances of the tryptic core peptides are low in comparison to the completely cleaved tryptic peptides (Fig. 4 and Supplementary Information: Fig. S6). It should be noted that LC-MS abundances are peptide specific depending on the ionisation response. A potentially poor ionisation response of the large peptides with ring structures could lead to underestimation of their abundance.

More detailed analysis revealed that for the predicted N-terminus of the NsoA1 core peptide most spectra matched the tryptic peptide (K)SACTPGCPTGILMTCPLK with 166 PSMs in total (Table 4). Also, the LC-MS abundance of this peptide was the highest of all detected tryptic peptides (Supplementary Information: Fig. S6). The N-terminal tryptic cleavage site (27 K↓28 S) is located inside the predicted ring A loop (Fig. 1a), and the efficient production of this tryptic peptide suggests that ring A is commonly not present. Furthermore, CAM modification of all three C residues was observed in 126 of the 166 PSMs of this peptide, excluding the presence of all three lanthionine rings. An additional 29 CAM modifications were detected on C(3), 12 on C(7) and 36 on C(15) resulting in 155 CAM on C(3), 136 on C(7), and 166 (all) on C(15) (Table 4 and Supplementary Information: Scaffold file 2). This left only a small number of PSMs with free C residues which could potentially have one of the lanthionine rings (B, C) formed. Inspection of the corresponding MS/MS spectra did not support the presence of rings (Supplementary Information: Fig. S7). A small number of tryptic peptides from the N-terminus of the core peptide produced by missed cleavages of trypsin were detected (see Table 3). Two out of the 6 spectra matching the peptide YKSKSACTPGCPTGILMTCPLK covering the whole ring A region would allow for the presence of ring A, but not rings B and C (Supplementary Information: Fig. S8). Taken together, the results for the N-terminal part of the NsoA1-3 core peptide indicate that in the majority of the peptides lanthionine rings A to C are not formed.

**Table 4:** List of PSMs and their modifications for the tryptic peptide SACTPGCPTGILMTCPLK.

		Dehydration											
	CAM	NONE	T4	T9	T4 + T9	T4 + T14	T9 + T14	T14	T4 + T9 + T14	S1	S1 + T14	S1 + T9 + T14	total
C3	3						2		1				3
C3 + C7	1							1					1
C3 + C15	25	0	7	2	0	7	3	0	1	0	2	3	25
C7 + C15	11	0	2	2	0	1	2	0		1	2	1	11
C3 + C7 + C15	126	68		3	24	2		29					58
Total PSMs	166	68	9	7	24	10	7	30	2	1	4	4	98

Residues have been numbered in this peptide sequence and the C residues are given in the first column. The column labelled CAM gives numbers of CAM modifications on the C-residues while the following columns show the distribution of dehydration on S and T residues. The last column (total) refers to total number of PSMs showing the dehydrations of the specified versions of CAM modified peptides.

### Examination of the core peptide C-terminus

**Figure 5:**
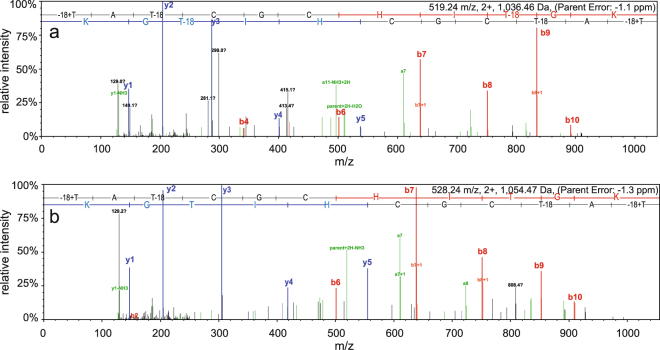
MS/MS spectra matching the C-terminal tryptic peptide TATCGCHITGK. For details see Fig. 3 legend. Spectra of PSMs with no CAM modification of C residues and with 3 (**a**) or 2 (**b**) dehydrations on T residues. Fragments can only be observed outside the putative ring area after residue C6, indicating the presence of both rings (see Fig. 1). For fragment masses see Tables S8 and S9.

The C-terminal tryptic peptide TATCGCHITGK covering the putative ring D and E regions was also observed with several different modifications (Table 5). The results show that in most of the observed PSMs for peptide TATCGCHITGK cysteine residues were modified by CAM, ruling out their engagement in ring formation. On the other hand, 27 out of the 123 PSMs for peptide TATCGCHITGK did not have any CAM modification and 18 of them were also fully dehydrated. The remaining 9 lacked dehydration of T(9), but since T(9) is not directly involved in ring formation, all 27 detected peptides could have rings D and E formed. Out of these 27, several corresponding MS/MS spectra clearly indicated the presence of rings D and E. This can be concluded from the absence of fragmentation in the ring region whereas the remaining linear part showed normal fragmentation (Fig. 5).

**Table 5:** List of PSMs and their modifications for the tryptic peptide TATCGCHITGK. For details see header of Table 4.

		Dehydration							
	CAM	NONE	T1 + T3	T1 + T9	T1 + T3 + T9	T3	T3 + T9	T9	total
C4	20					13	6	1	20
C4 + C6	41	21					3	17	20
C6	35			1		15	18	1	35
all C unmodified			9		18				27
Total PSMs	123

The LC-MS chromatograms show that the different versions of the peptide TATCGCHITGK elute at different retention times (Fig. 4). The peak for TATCGCHITGK with 1 CAM and 2 dehydrations appears as the strongest one while the fully dehydrated version without any CAM appears as the weakest peak. The latter version elutes in two peaks which could be the forms with and without rings (Fig. 4b). The fully dehydrated version of the YK tryptic core peptide also eluted as two peaks potentially presenting a separation in peptides with and without rings (Fig. 4b). Based on spectrum count (Table 5), approximately 22% of the detected TATCGCHITGK peptides undergo formation of rings D and E in the used expression system.

### Summary

In summary, the results presented here show that uncleaved preNsoA peptides are generated in the heterologous expression system. The majority of the preNsoA peptides seem to be incompletely modified. However, a certain amount of completely modified preNsoA1-3 peptides is also generated. Despite the incomplete processing, strong antimicrobial activity has been observed in overlay assays in the presence of trypsin^30^. Trypsin cleaves preNosA1-3 in the region of the predicted cleavage site for the leader peptide removal and could produce a certain amount of leaderless modified active NsoA1-3 peptide as observed in the LC-MS analyses. According to the LC-MS data, relatively large amounts of fully cleaved tryptic peptides should be generated in the activity assays in addition, some of which would still carry modifications and ring structures. Antibacterial lantipeptides are believed to exert biological activity by inhibiting cell wall biosynthesis and/or disrupting membrane integrity through pore formation^8,43^. In several cases, the essential cell wall precursor lipid II serves as the target of the lantibiotic; binding to lipid II inhibits the transglycosylation reaction necessary to synthesize peptidoglycan and also sequesters lipid II into nonfunctional locations. Once bound to lipid II, nisin is able to insert into the membrane and form stable pores consisting of eight nisin and four lipid II molecules. New modes of action of lantibiotics have been suggested by results showing that nisin can be active in the absence of lipid II^44^; at high concentrations, nisin non-specifically interacted with phospholipids in lipid-II deprived membranes leading to membrane destabilisation and inhibition of bacterial growth. Previous work showed that different regions of the nisin molecule contribute to its various activities – for example, removal of rings D and E prevented target membrane permeabilization but did not prevent antimicrobial activity^45^. This suggests that some shorter N-terminal Nso tryptic peptides might contribute to the observed antimicrobial effect. However, formation of rings A and B, which interact with lipid II^46^, and ring C which has been shown to be crucial for full nisin A activity^47^, are likely to be essential. The low levels of ring formation in the Nso peptides may be an artefact of the heterologous expression – nisin A production is thought to be mediated by a multimeric lanthionine synthetase complex located at the membrane^10^, if an analogous system is used by the Nso peptides there could be differences in the heterologous host that impact upon complex formation or activity, and the absence of a suitable protease may also have an effect.

Several other lantibiotic peptides have been expressed in heterologous systems and differently modified peptides with antimicrobial activity have been observed. For example, when two class II lantibiotics were expressed by the nisin (class I) biosynthetic machinery, peptides with 1–6 dehydrations were observed; the 4–5-fold dehydrated peptides were separated and their activity was not significantly different from the mixture of the multifold dehydrated peptides^48^. Recently, the expression of 54 lantibiotic peptides in the nisin system was reported, and 5 of them were found to be active^24^. Interestingly, the expression of one of them, named flavucin, resulted in a heterogenous dehydration state; the different dehydration states were separated, and it was found that a truncated 5-fold dehydrated version had the highest activity against selected bacteria while other dehydrated forms performed better against other strains. It was suggested that such variation could occur naturally and provide the advantage of different antimicrobial spectra^24^.

High antimicrobial activity of NsoA was demonstrated in the presence of trypsin against Gram-positive gut pathogens such as C*. difficile*, a major cause of hospital acquired infections, and *C. perfringens* as well as against *L. lactis* MG1614^30^. Therefore, Nso has the potential to be developed as a novel clinical antimicrobial peptide active against infections with multidrug resistant bacteria. However, like many novel bacteriocin gene clusters, production in the native strain is problematic^49^. Heterologous expression and application of trypsin, MS and immunoprecipitation can allow analysis of lantibiotic modification and enable the production of active antimicrobials from genetic information.

## Material and Methods

### Preparation of protein extracts

The *L. lactis* strain UKLc10^50^ carrying the *nso* gene cluster was used to produce Nso peptides^30^. The *nso* operon on vector pIL253 (p*nso*) was either present alone or in combination with a second plasmid pTG*nsoA3*_*nsoA4*, containing the gene sequences for *nsoA3–4* under the control of the nisin A promoter in vector pTG262^30^. Details of the expression of the *nso* cluster in *L. lactis*, induction with nisin, preparation of protein extracts, SDS-PAGE electrophoresis, and Western blotting were described previously^30^. For Western blotting, a polyclonal anti-leader peptide antibody raised by Genscript Corp (NJ, USA) from synthesized N-terminal acetylated NsoA2 leader peptide with a C-terminal cysteine (H_2_N-AKFDDFDLDVTKTAAQGGC-CONH_2_) was used with anti-Rabbit IgG-alkaline phosphatase (Sigma) as the secondary antibody^30^.

### Immunoprecipitation

Immunoprecipitation was performed using the Pierce™ Crosslink IP Kit (Thermo Fisher Scientific) according to the manufacturer’s protocol. In brief, 1 ml of slurry (500 μl agarose beads) was adjusted to 10 ml with coupling buffer (0.01 M sodium phosphate, 0.15 M NaCl; pH 7.2, from kit) and 2 ml of the NsoA2 leader peptide antibody solution (60 mg protein) and incubated at 4 °C for 2 h on a vortex mixer. The beads were removed and washed with coupling buffer, then incubated with the DSS cross-linker for 60 min, and washed with elution buffer (pH 2.8, from kit). The beads were then equilibrated with 25 mM Tris-HCl buffer, pH 7.4, and incubated with 20 ml of the cell extract (15 mg ml^−1^ protein) in the same buffer on a vortex mixer at 4 °C overnight. Beads were washed with 25 mM Tris-HCl buffer, pH 7.4, and bound peptides eluted in 4 sequential steps with 0.5 ml elution buffer each. The fractions were neutralized with 1 M Tris-HCl pH 9.5, and aliquots used for LC-MS analysis.

### LC-MS analysis of IP samples and gel slices

Intact preNsoA1-3 peptide prepared by immunoprecipitation was characterised by LC-MS on a Synapt G2-Si mass spectrometer coupled to an Acquity UPLC system (Waters, Manchester, UK). Aliquots of the samples were injected onto an Aeris WIDEPORE 3.6 µ C4 column (Phenomenex, Macclesfield, UK) and eluted with a gradient of 1–95% acetonitrile (Acquity curve 7) water/0.1% formic acid in 14 min with a flow rate of 0.2 ml min^−1^. The mass spectrometer was controlled by the Masslynx 4.1 software (Waters) and operated in positive MS-ToF and resolution mode with a capillary voltage of 2.5 kV and a cone voltage of 40 V in the range of *m/z* 50–1600. Leu-enkephalin peptide (1 ng ml^−1^, Waters) was infused at 10 µl min^−1^ as a lock mass and measured every 30 s. Spectra were generated in Masslynx 4.1 by combining several scans, and the spectra were deconvoluted using the MaxEnt3 tool in Masslynx. To alkylate free cysteine residues in IP generated preNsoA1-3, aliquots were at first incubated with 5 mM DTT (65 °C, 30 min). Iodoacetamide (IAA) was added to 10 mM and incubation continued at room temperature (30 min).

Gel slices containing a 6 kDa band reacting with the anti-Nso leader peptide antibody were prepared according to standard procedures^51^. Briefly, the slices were washed with 50 mM TEAB buffer pH 8 (Sigma), incubated with 10 mM DTT for 30 min at 65 °C followed by incubation with 20 mM iodoacetamide (IAA) at room temperature (both in 50 mM TEAB). After washing and dehydration with acetonitrile, the gels were soaked with 50 mM TEAB containing 10 ng/µl Sequencing Grade Trypsin (Promega) and incubated at 50 °C for 8 h. Aliquots were analysed by nanoLC-MS/MS on an Orbitrap Fusion™ Tribrid™ Mass Spectrometer coupled to an UltiMate^®^ 3000 RSLCnano LC system (Thermo Scientific, Hemel Hempstead, UK). The sample was separated on a PepMap™ 100 C18 LC Column (C18, 2 µm, 500 × 0.75 mm, Thermo) using a gradient of 0.75% min^−1^ acetonitrile from 6% to 40% in water/0.1% formic acid at a flow rate of 0.3 µl min^−1^ and infused directly into the mass spectrometer. The mass spectrometer was run in positive ion mode with quad isolation at 120 K resolution over the mass range *m/z* 400–1600 for the precursor scans (orbitrap). One microscan of 50 ms with an AGC target of 2e^5^ was used. MS2 threshold was set to 2e^4^ and precursors fragmented by both CID and HCD with CE = 30 and an isolation window of 1.6 Da (quadrupole) using the automatic maximum speed option with ion injection for all available parallelisable time. Dynamic exclusion was set to 1 count and 40 s. Recalibrated peaklists were generated using MaxQuant 1.5.3.30^52^, and the database search was performed with the merged HCD and CID peaklists using Mascot 2.4.1 (Matrixscience, London, UK). The search was performed on a *Lactococcus lactis* protein sequence database (Uniprot, October 2017, 2,229 sequences) to which the preNsoA peptide sequences had been added with a precursor tolerance of 6 ppm and a fragment tolerance of 0.6 Da. The enzyme was set to trypsin/P with a maximum of 2 or 3 allowed missed cleavages. Dehydration (−18) of serine and threonine, oxidation of methionine and carbamido-methylation (CAM) of cysteine were set as variable modifications. The Mascot search results were imported into Scaffold 4.4.1.1 (www.proteomsoftware.com) using identification probabilities of 99% for proteins and 95% or 0% for peptides, as discussed in the results. Annotated spectra generated in Scaffold were exported for presentation (Figures). Scaffold files with detailed database search results are provided as Supplementary Information. The software is freely available as a viewer.

### Data availability

The mass spectrometry proteomics data have been deposited to the ProteomeXchange Consortium via the PRIDE^53^ partner repository with the dataset identifier PXD008372.

## Electronic supplementary material

Supplementary Information
