# Supplementary material for: Processing and Structure of the Lantibiotic Peptide Nso From the Human Gut Bacterium Blautia obeum A2-162 analysed by Mass Spectrometry
Source: Sci Rep. 2018 Jul 4;8:10077. doi: 10.1038/s41598-018-28248-6 (PMC6031655; doi:10.1038/s41598-018-28248-6)
Supplement: Supplementary file 1 — Supplementary Information [file 41598_2018_28248_MOESM1_ESM.pdf]

Supplementary Information

Processing and Structure of the Lantibiotic Peptide Nso From the Human Gut Bacterium *Blautia obeum* A2-162 analysed by Mass Spectrometry

Cristina Gherghisan-Filip[1†+], Gerhard Saalbach*[2†], Diane Hatziioanou[1], Arjan Narbad[1], Melinda J. Mayer*[1]

[1]Quadram Institute Bioscience, Gut Microbes and Health Institute Strategic Programme, Norwich Research Park, Norwich, NR4 7UA, UK, [2]John Innes Centre, Department of Biological Chemistry, Norwich Research Park, Norwich, NR4 7UH, UK,
[†]these authors contributed equally to this work
*corresponding authors
[+]Present Address: University of East Anglia, UEA, Norwich Medical School, Norwich, NR4 7TJ

a

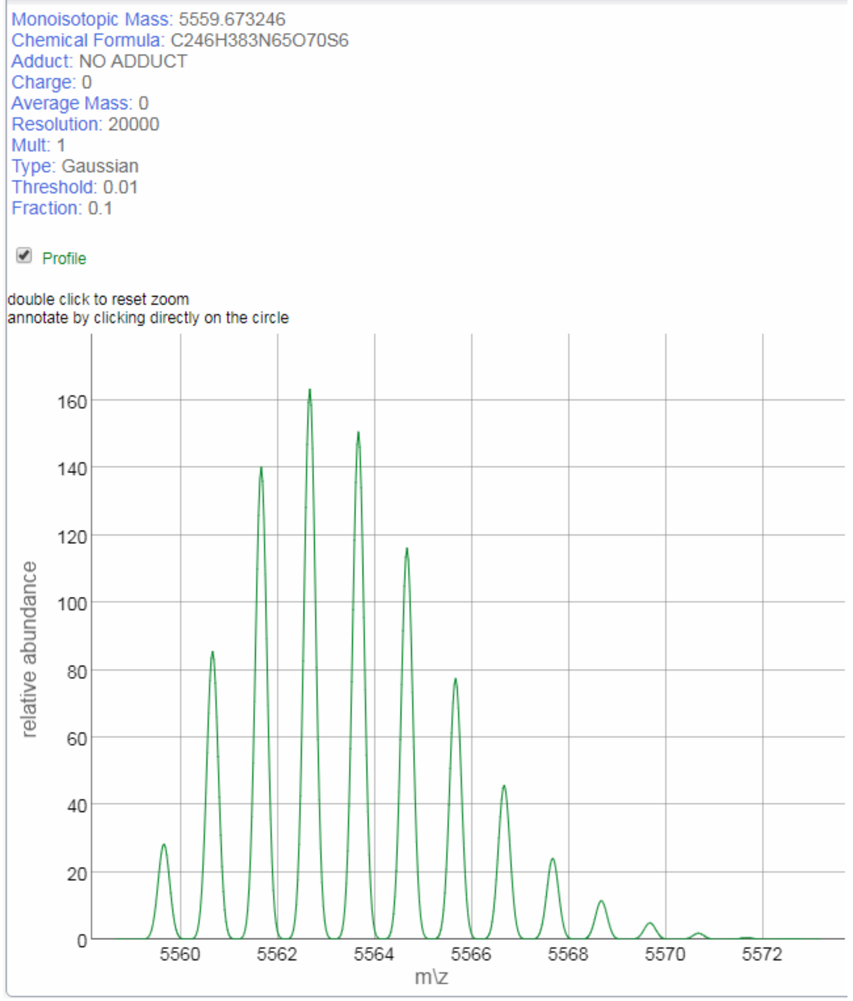

b

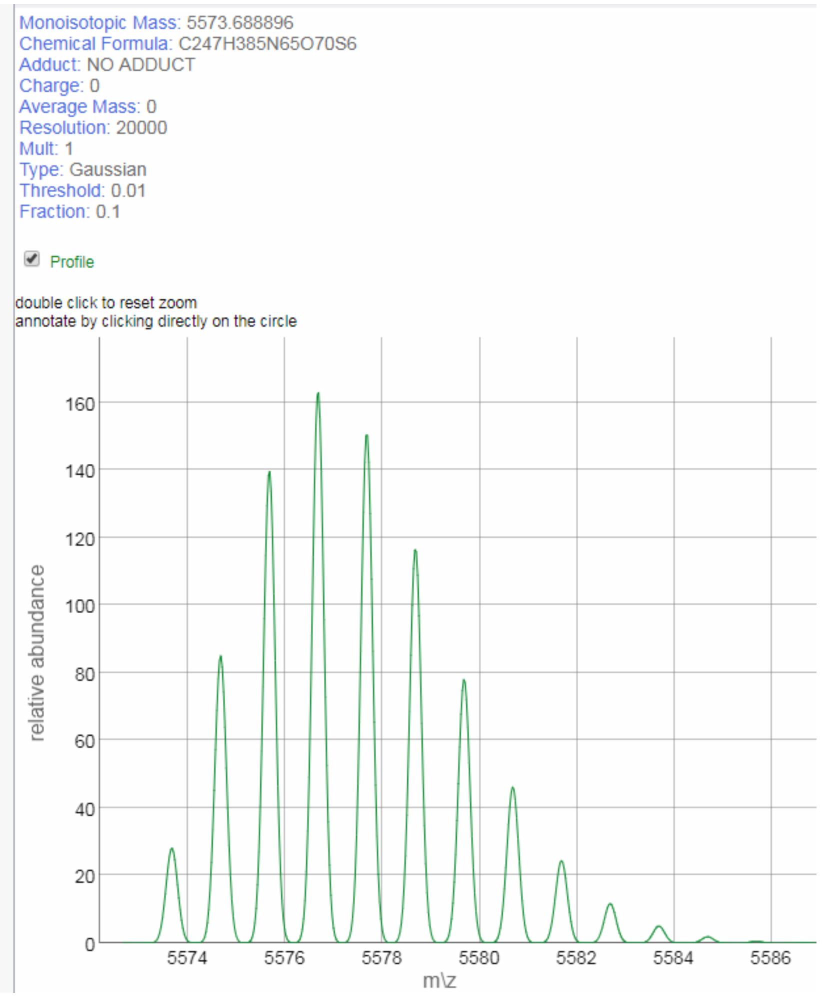

Supplementary Information Fig. S1:

Predicted isotope patterns of preNsoA1-8H_2O (a) and preNsoA2-3-8H_2O (b). A comparison of the patterns of preNsoA2-3 with the observed pattern is shown in (c-d) (next slide)

The patterns are very similar since there is only a single residue difference between preNsoA1 and preNsoA2-3

The predicted patterns were calculated using http://www.envipat.eawag.ch without adduct/charge

c

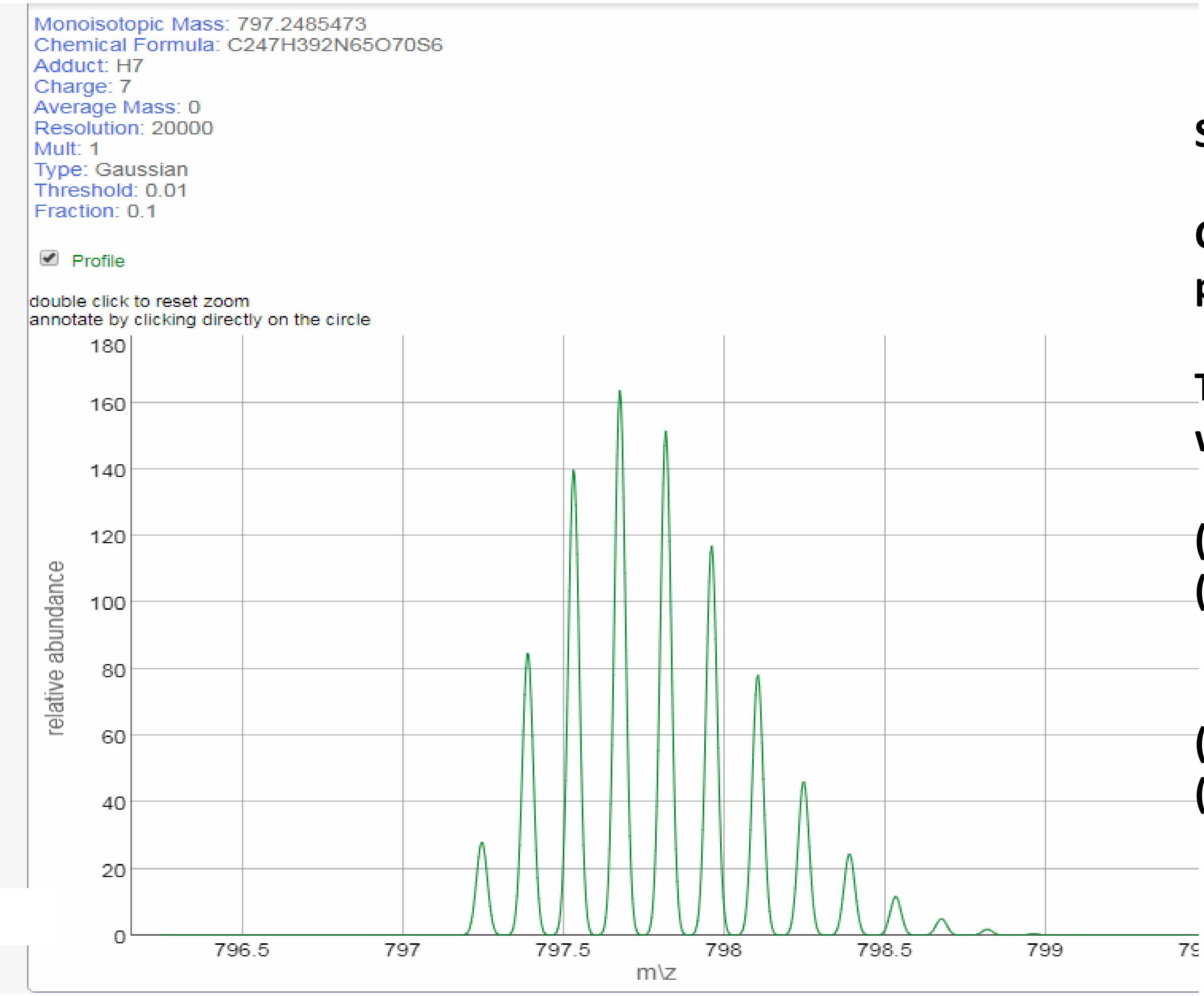

d

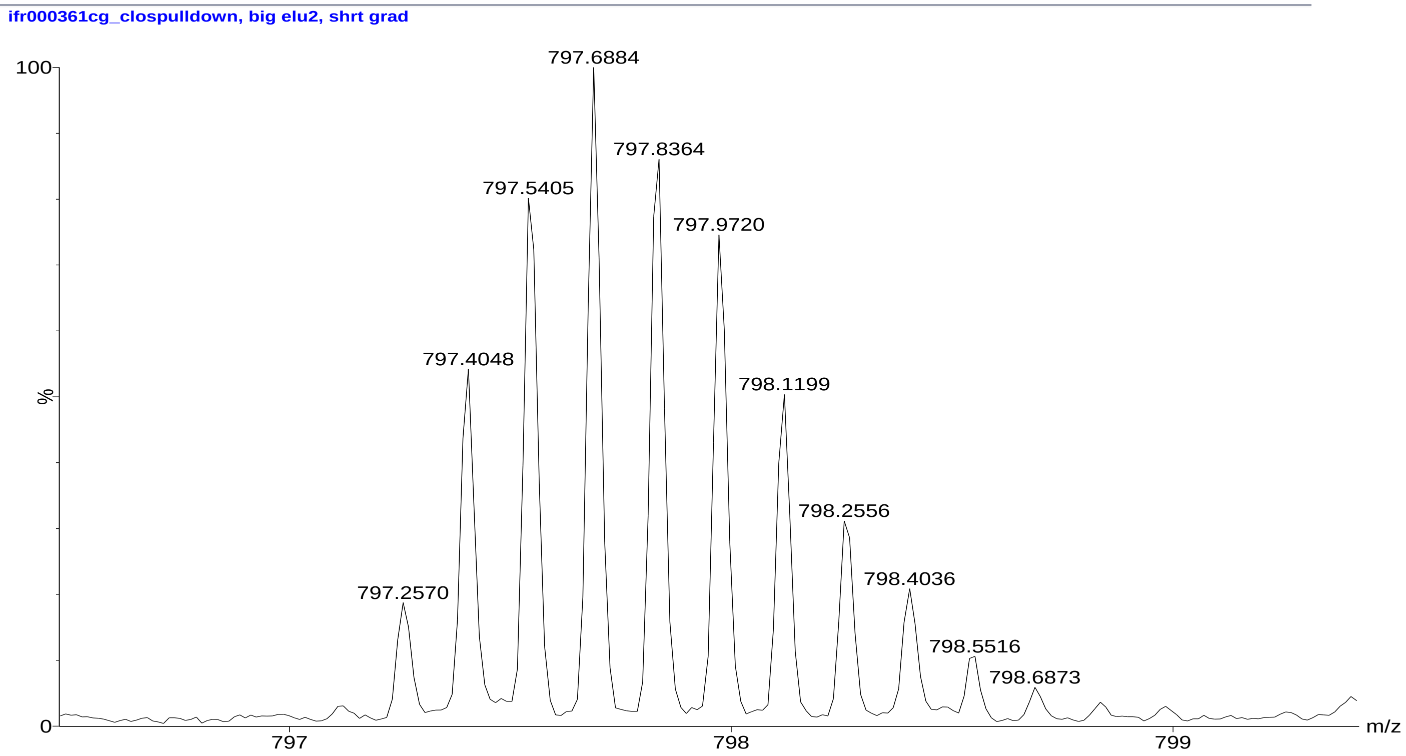

Supplementary Information Fig. S1 (continued)

Comparison of the predicted isotope pattern of preNsoA2-3 with the observed pattern.

The observed isotope pattern of preNsoA1 is too weak for a reliable comparison (see Fig. S2a)

(c) Predicted isotope pattern of preNsoA2-3 (charge 7+) http://www.envipat.eawag.ch

(d) Observed isotope pattern of preNsoA2-3 (charge 7+)

Supplementary Information Fig. S2a:

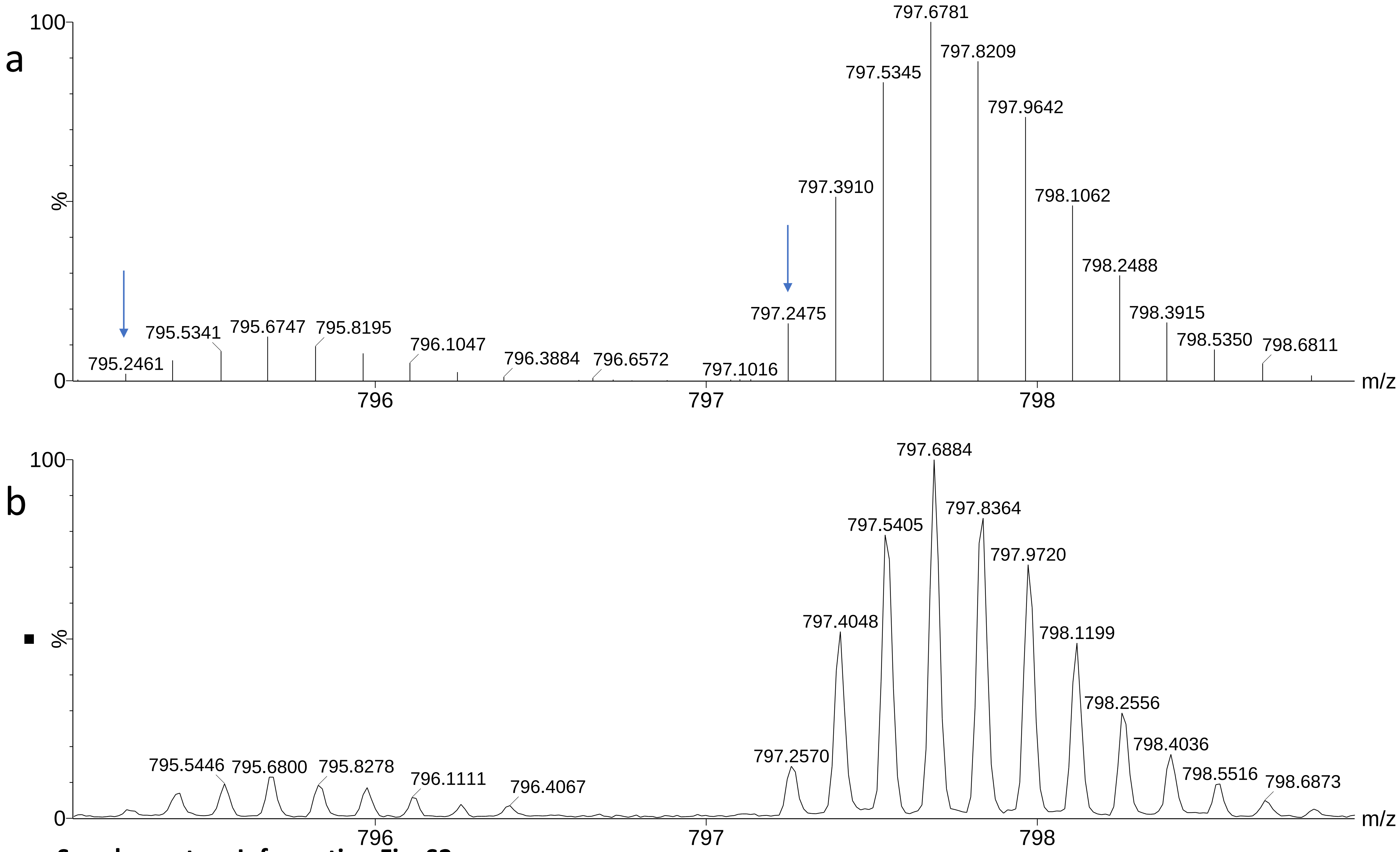

Supplementary Information Fig. S2a:

Example for calculating the accurate measured mass for preNsoA1 – 8H_2O (A1) and preNsoA2/3 – 8H_2O (A2).

The sample was analysed by LC-MS on the Synapt G2-Si mass spectrometer (Waters) with Leu-enkephalin as lock mass (*m/z* 556.2766).

(a) Lock mass corrected isotope patterns at charge state 7+ showing the monoisotopic peaks at *m/z* 795.2461 (A1) and *m/z* 797.2475 (A2) (arrows) corresponding to deconvoluted monoisotopic masses of 5559.6718 (A1) and 5573.6816 (A2) or *m/z* of 1+ of 5560.6791 and 5574.6889.

(b) Acquired raw spectrum without lock mass correction

Supplementary Information Fig. S2b:

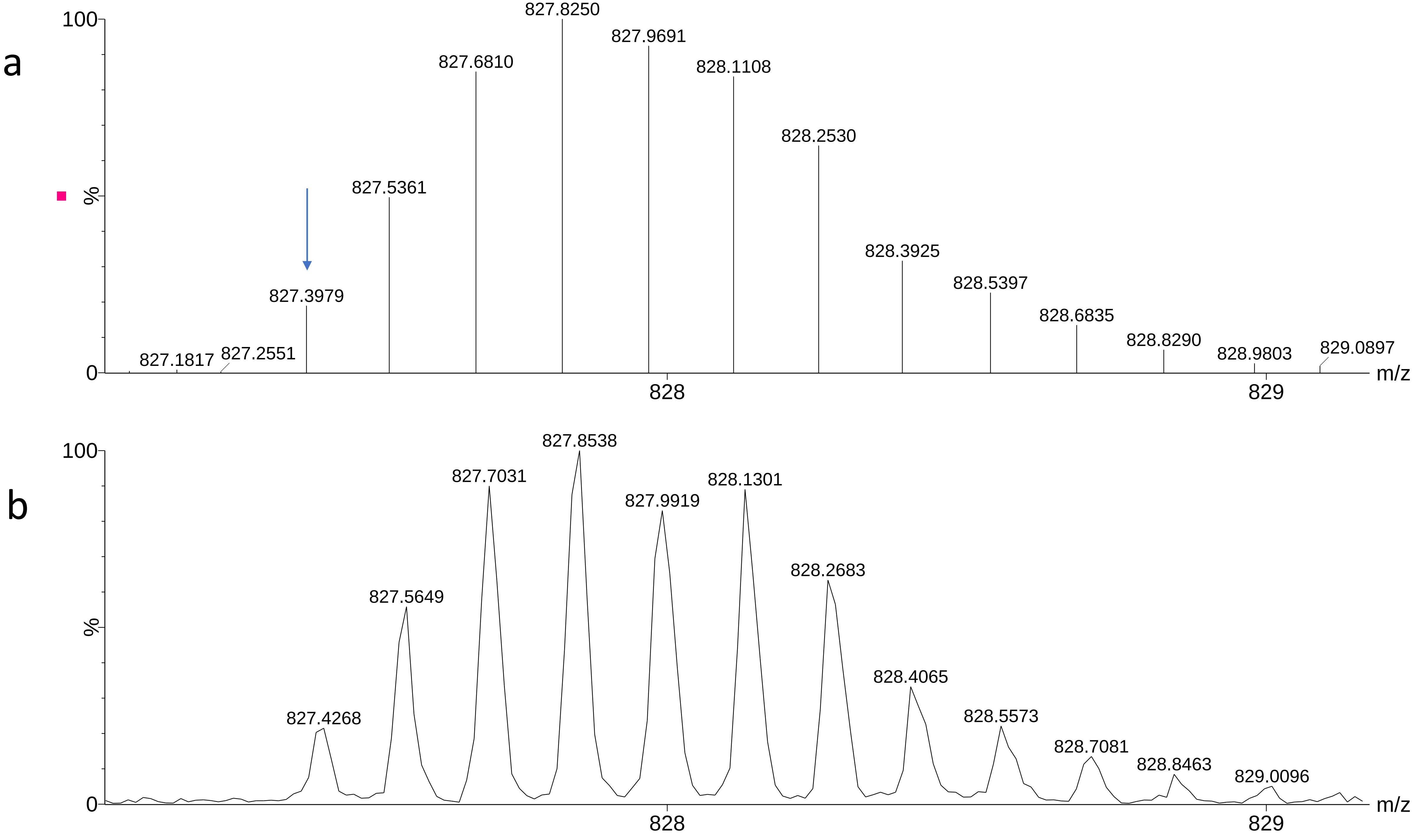

Supplementary Information Fig. S2b:

Example for calculating the accurate measured mass for IAA-treated preNsoA1 – 5H_2O.

The sample was analysed by LC-MS on the Synapt G2-Si mass spectrometer (Waters) with Leu-enkephalin as lock mass (*m/z* 556.2766).

(a) Lock mass corrected isotope pattern of preNsoA1 – 5H_2O + 3 CAM at charge state 7+ showing the monoisotopic peak at *m/z* 827.3979 (arrow) corresponding to a deconvoluted monoisotopic mass of 5784.7344 or *m/z* 5785.7417 (1+). The theoretical mass is 5784.7691 (*m/z* 5785.7764) and the error 6 ppm.

(b) Acquired raw spectrum without lock mass correction

Supplementary Figure S3

LC-MS spectra of IP generated preNsoA peptides.
(a) MS spectrum showing charge states 4+ to 9+ of preNsoA peptides
(b,c) Zoom of the isotope pattern of charge state 7+ from 2 different experiments. After deconvolution (see Fig. 1 main text) the isotope patterns can be assigned to fully dehydrated preNsoA1 and preNsoA2/3 (most abundant) and a series of patterns indicating the presence of lower dehydration states.

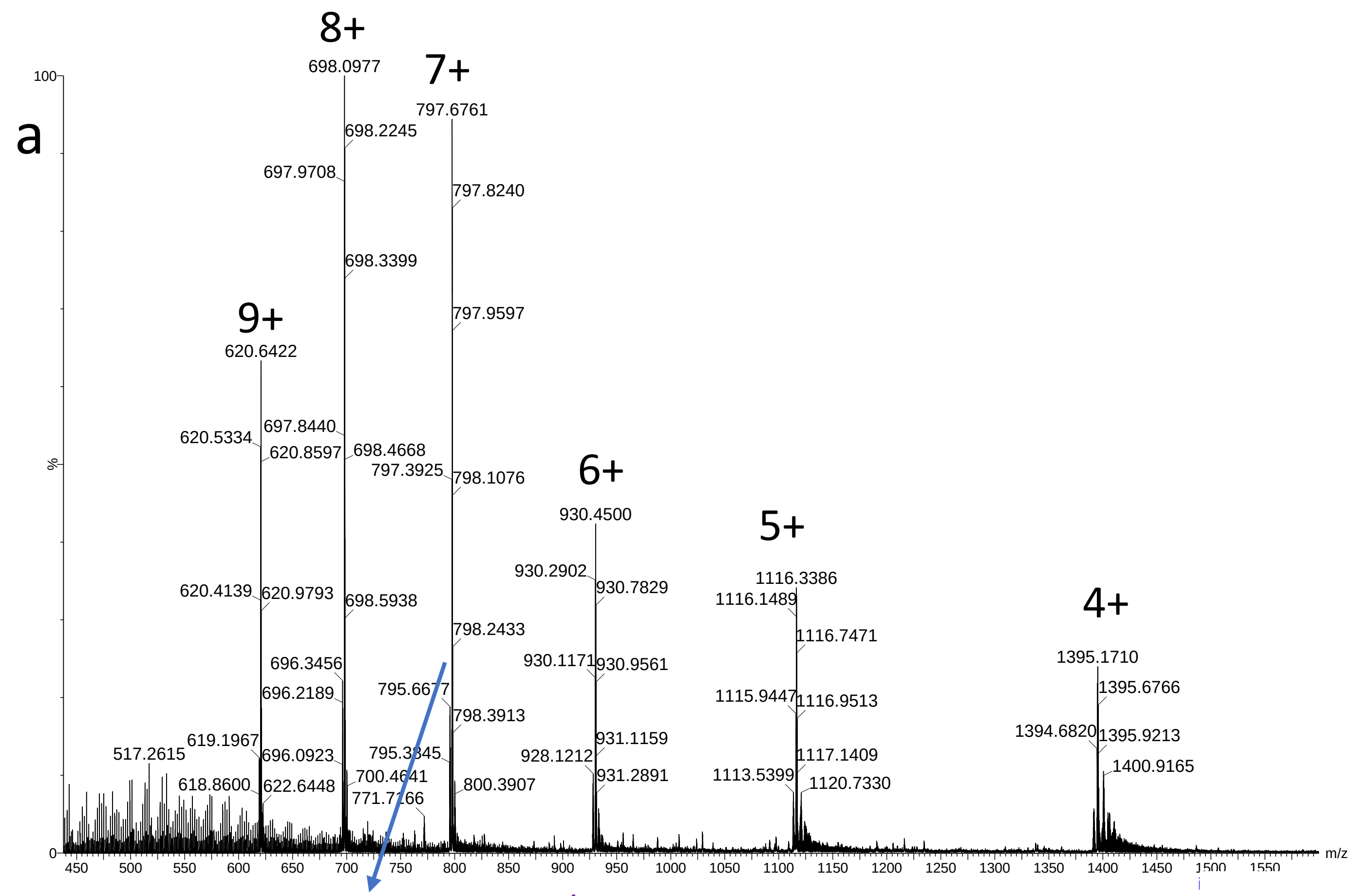

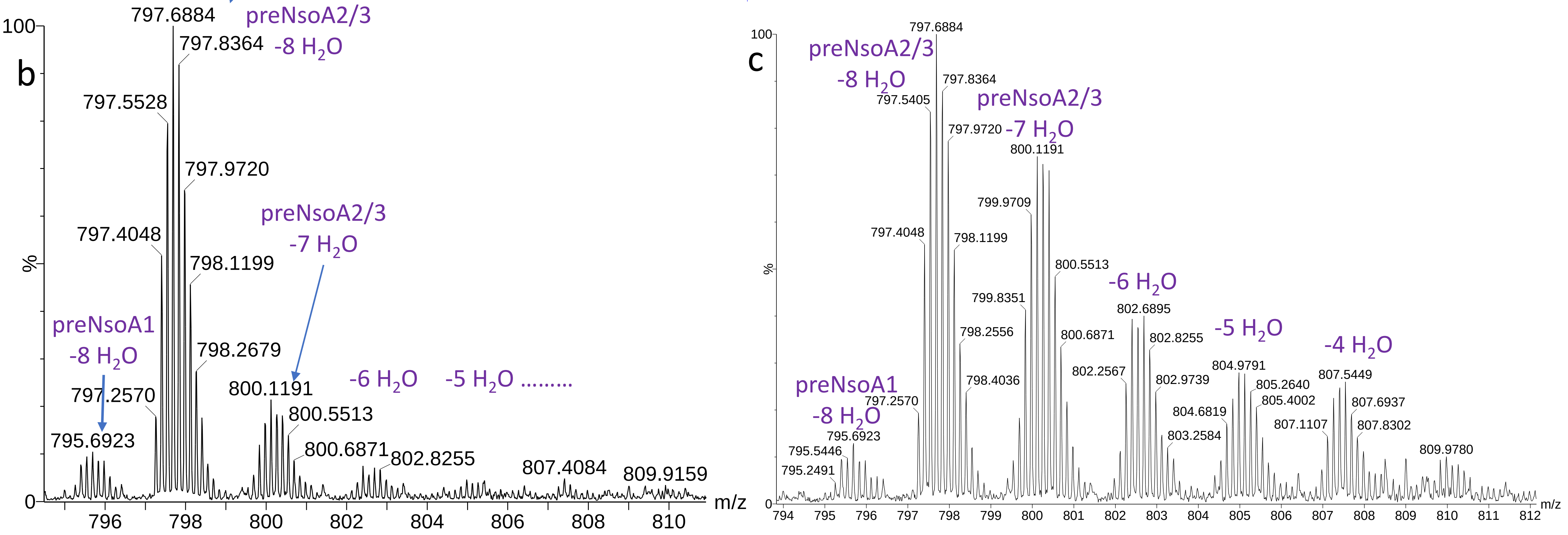

Supplementary Figure S4a

LC-MS spectrum of IP generated preNsoA1-3 peptides treated with iodoacetamide for alkylation of free cysteine residues.

Spectra were combined over a range of 0.4 min where all versions of the modified preNsoA1-3 peptides eluted. Monoisotopic masses (for zoom see Figure S4b) were selected and deconvoluted manually resulting in values shown in Table 1 (main text). Some modified peptide versions are indicated.

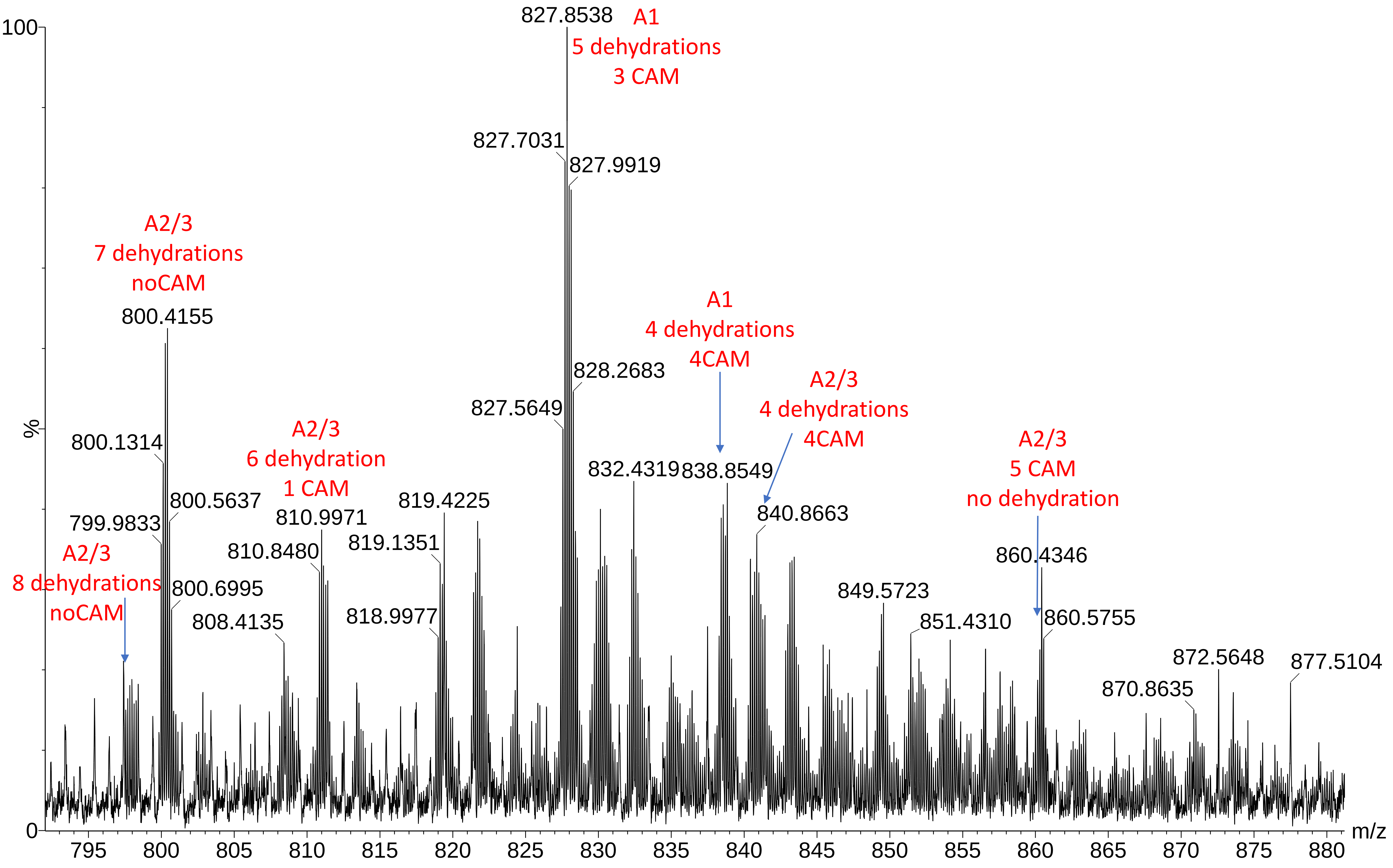

Supplementary Figure S4b

LC-MS spectra of IP generated preNsoA peptides treated with iodoacetamide for alkylation of free cysteine residues.

Monoisotopic masses were selected manually from all detectable isotope patterns in the range shown in Fig. S4a and deconvoluted resulting in values shown in Table 1. The most abundant isotope pattern (*) at *m/z* 827 could be assigned to preNsoA1 – 5 H_2O + 3 CAM indicating incomplete dehydration and incomplete ring formation.

However, as shown in Fig. S4c and Table 1, after deconvolution a series of dehydration and CAM modification states of both preNsoA1 and preNsoA2/3 can be assigned

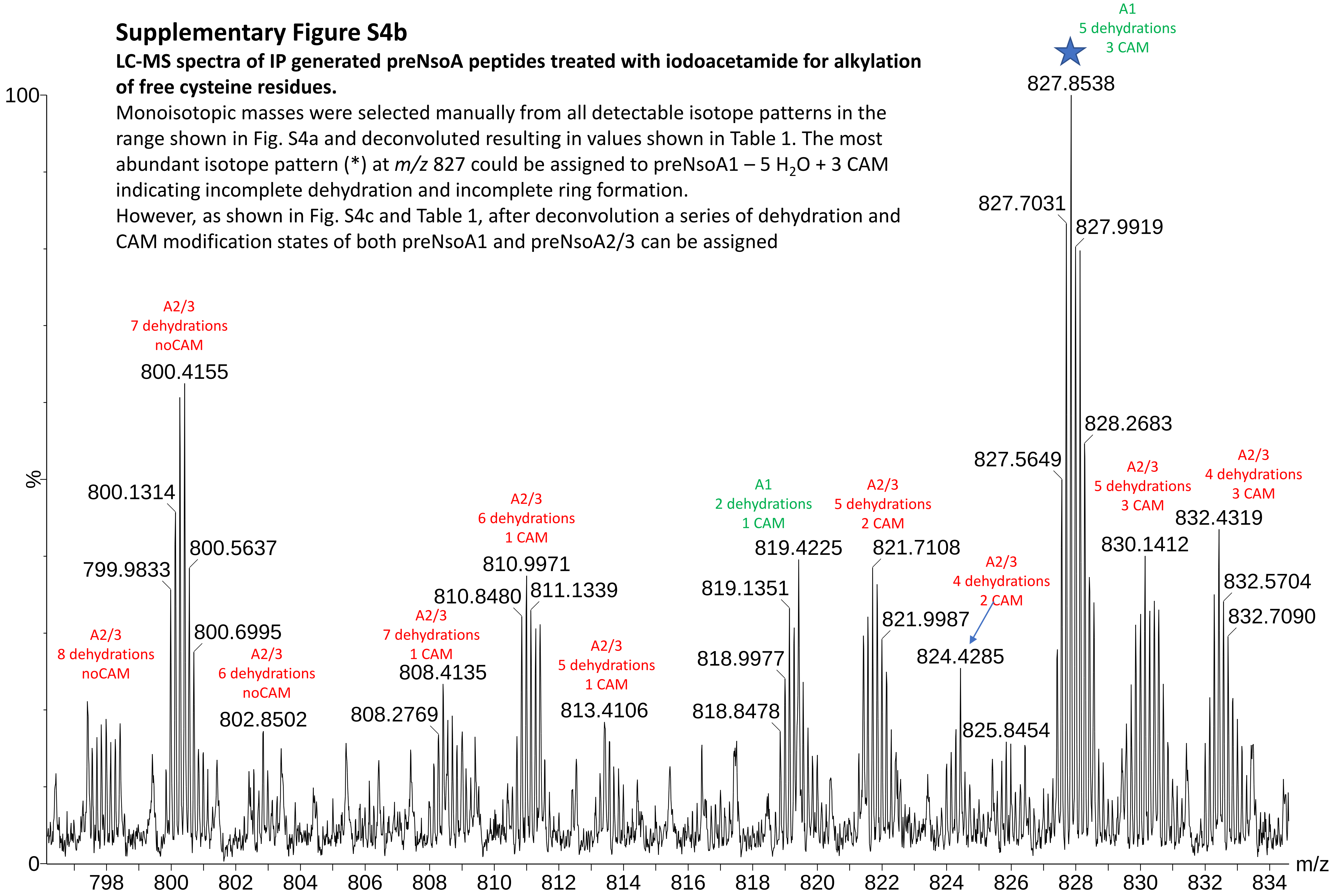

Supplementary Figure S4c:

Spectrum of IAA treated preNsoA peptide deconvoluted from a selected range of isotope patterns using the MaxEnt3 tool in Masslynx (Waters):
The spectrum was deconvoluted from charge state 8 using the MaxEnt3 tool in Masslynx. Masses are given as *m/z* of charge state 1+.
It shows a portion of 7- and 8-fold dehydrated preNsoA2/3 with no CAM modification (*) indicating complete modification including formation of all 5 rings.
Otherwise, a predominant peak of preNsoA1 and several peaks of preNsoA2/3 with 3 to 5 CAM modifications are present indicating incomplete modification.

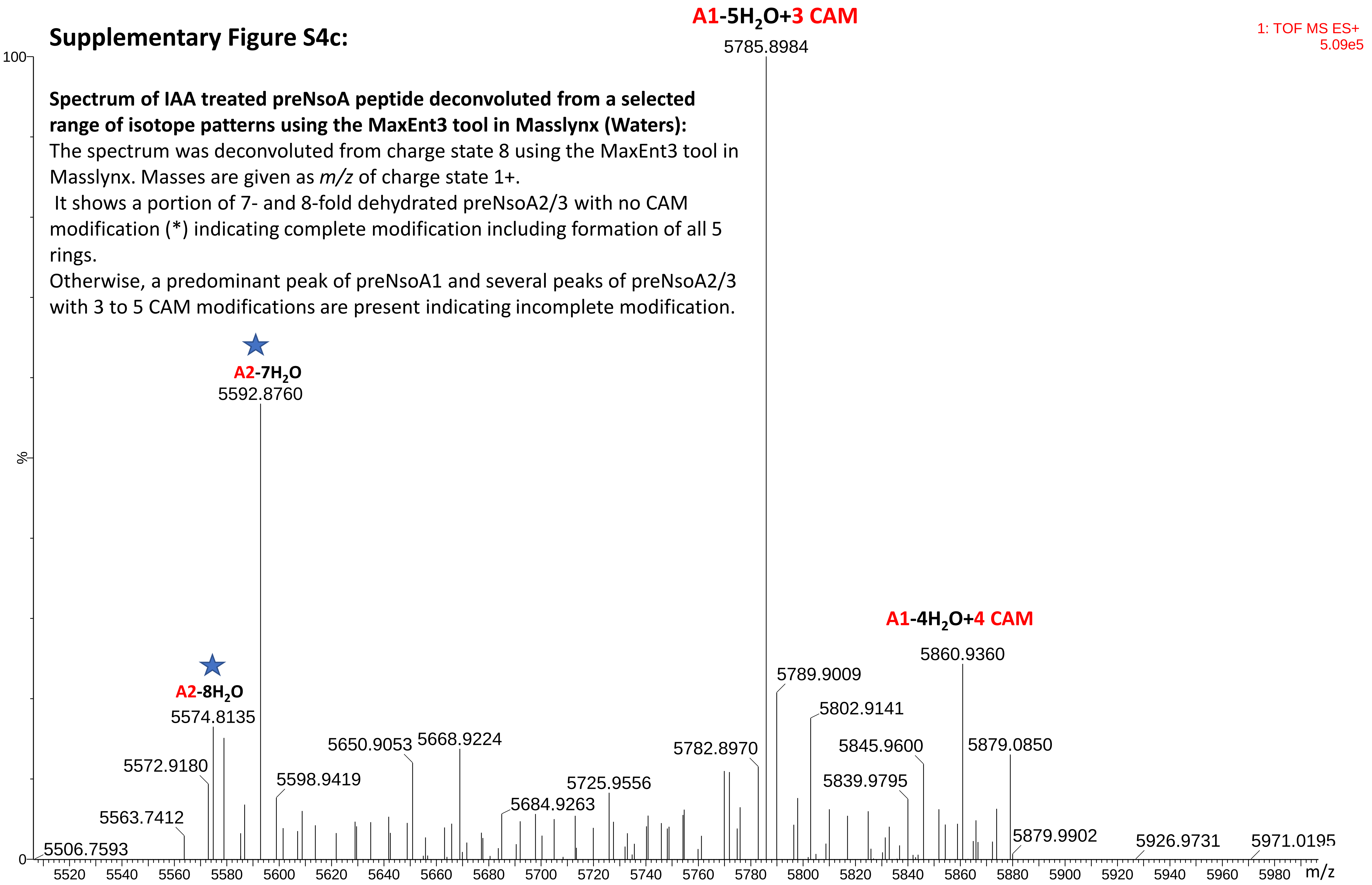

Supplementary Figure S5:

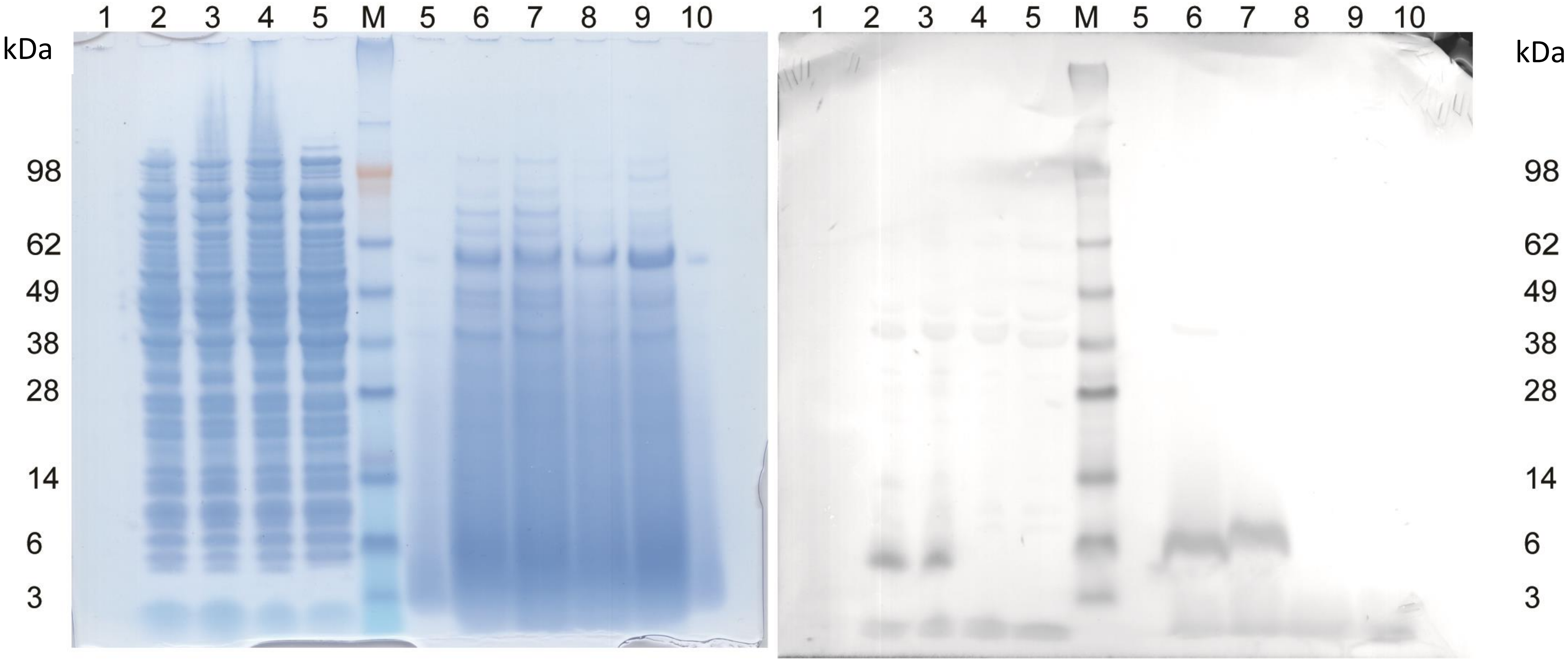

Supplementary Figure S5: SDS-PAGE and Western blot of protein extracts

Protein extracts are from cells (lanes 2-5) and TCA-precipitated supernatants (lanes 6-9) from *L. lactis* UKLc10 containing p*nso* (lanes 2, 6), p*nso* and pTG*nsoA3-nsoA4* (lanes 3, 7), p*nsoΔA* with *nsoA1-4* deleted[30] (lanes 4, 8) or the empty pIL253 vector (lanes 5, 9). M, SeeBlue Plus2 marker (Invitrogen). Lanes 1, 5, 10 no sample loaded. A band can be observed in the 6 kDa region in lanes 2,3,6,7.
Bands in the high mass range result from unspecific interaction with very abundant proteins.

Supplementary Figure S6:

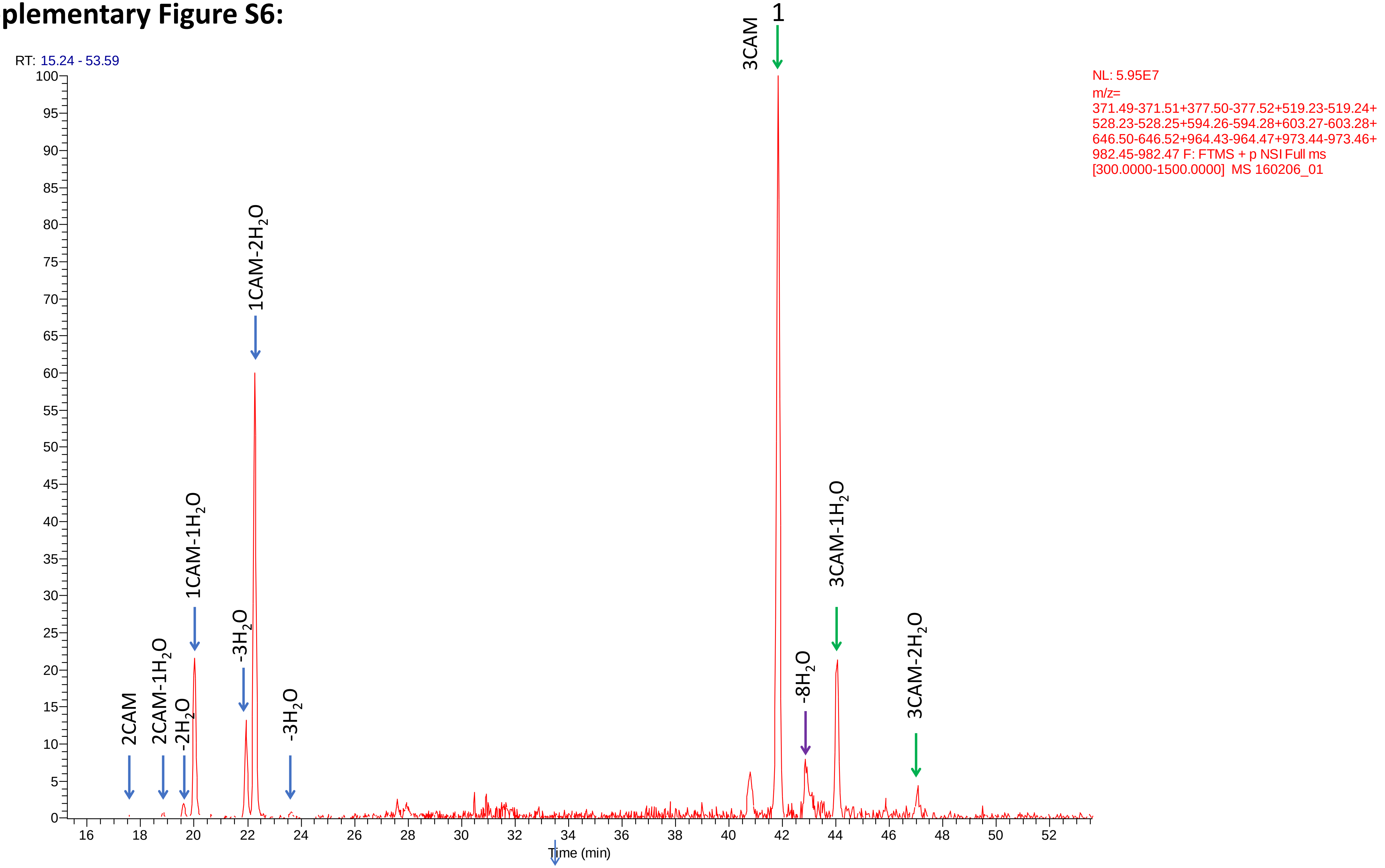

Supplementary Figure S6:

LC-MS elution profiles of tryptic peptides generated from preNsoA1-3

1: blue arrows: differently modified tryptic peptide TATCGCHITGK

2: green arrows: differently modified tryptic peptide SACTPGCPTGILMTCPLK

3: purple arrow: YKSKSACTPGCPTGILMTCPLKTATCGCHITGK-8H_2O; the 2nd peak for this peptides as shown in Fig. 4 (main text) overlaps with the strong green labelled peak 1

Supplementary Figure S7

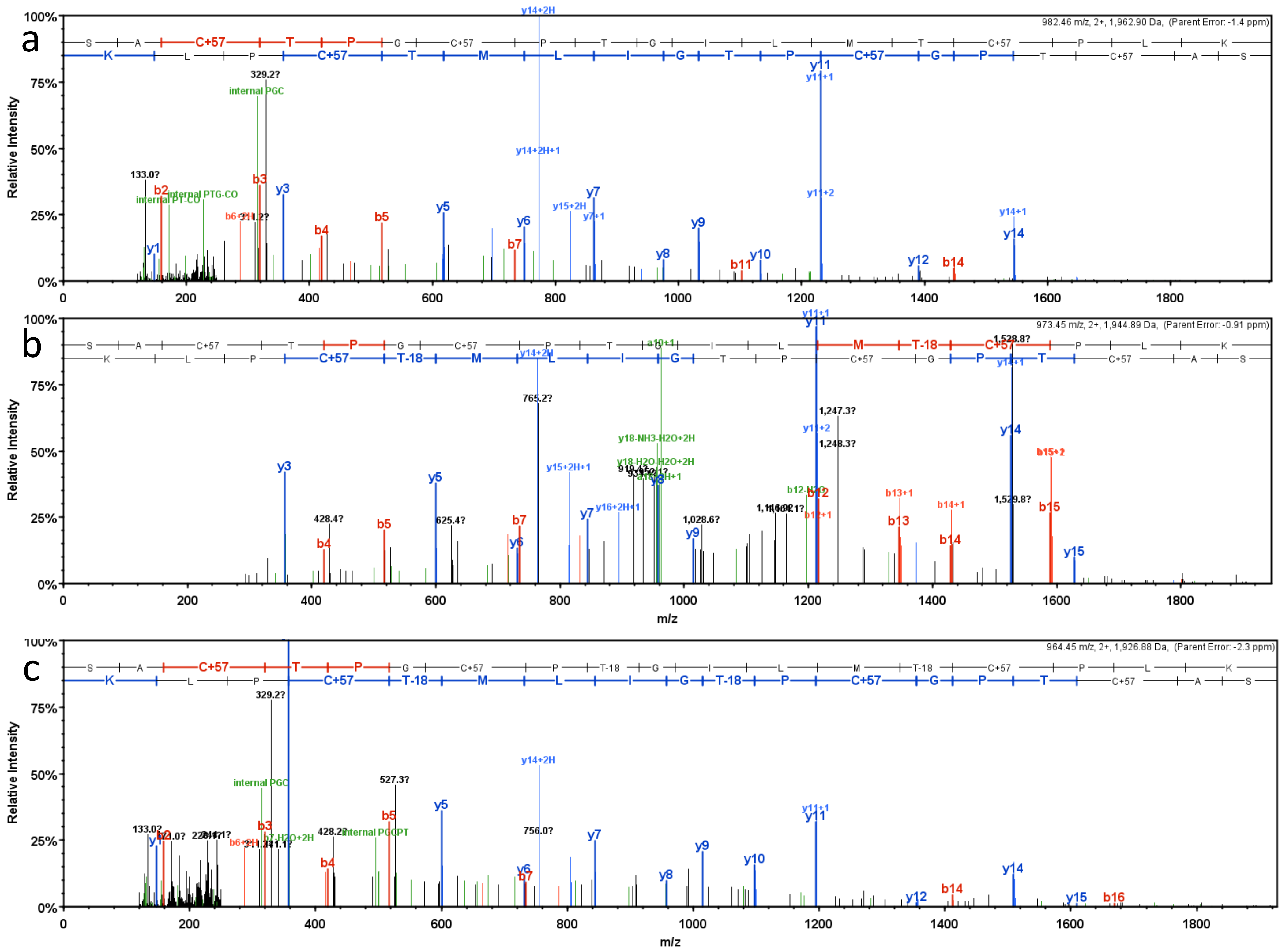

Supplementary Figure S7:

Selected MS2 spectra for the tryptic peptide SACTPGCPTGILMTCPLK from preNsoA1-3.
Although dehydrations are present (b,c), the series of fragment ions covering all ring regions indicate that no ring formation has occurred.

Supplementary Figure S8:

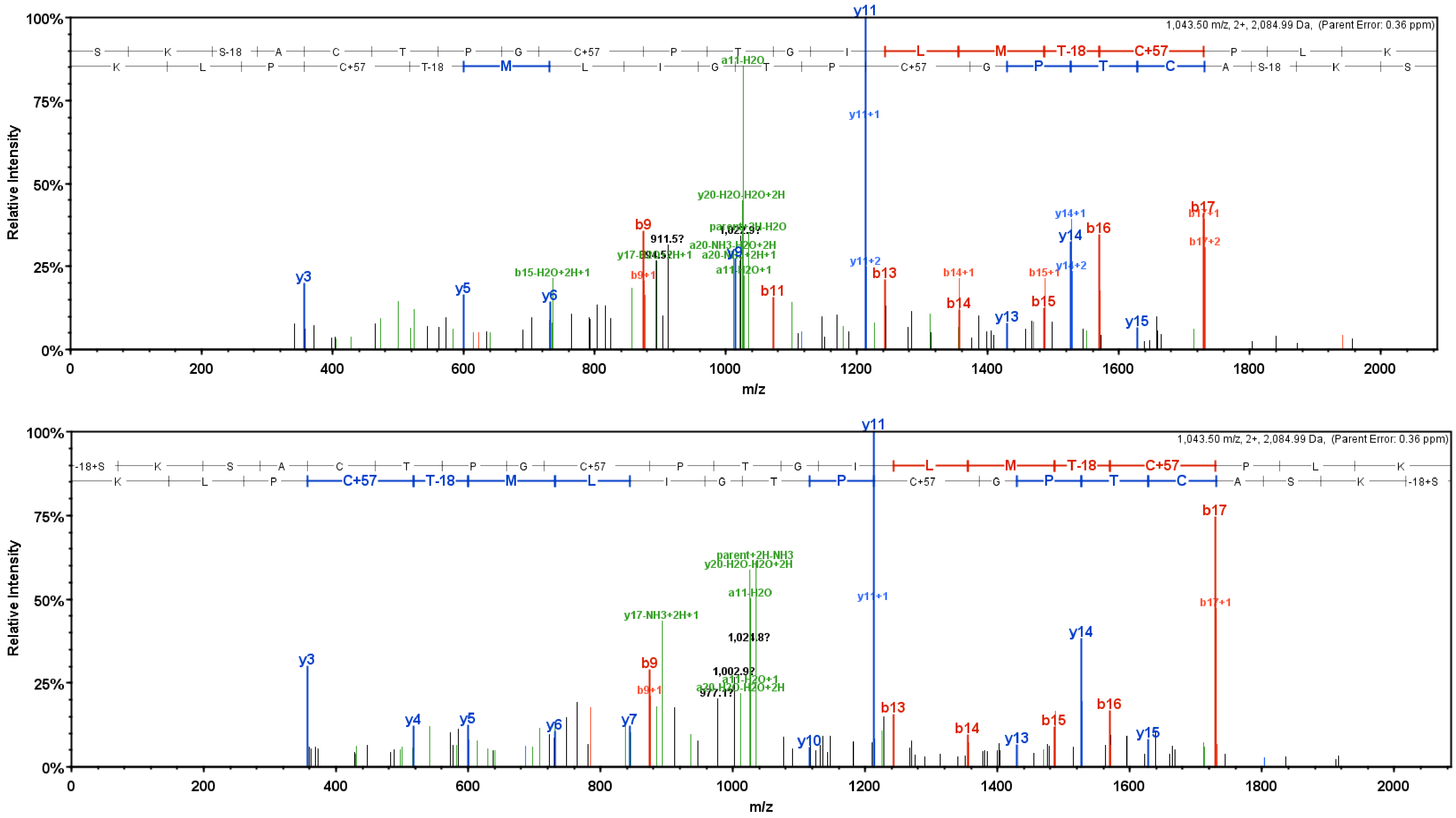

Supplementary Figure S8:

MS2 spectra for 2 selected PSMs matching the tryptic peptide SKSACTPGCPTGILMTCPLK from preNsoA1-3.

The presence of y-ions y11 to y15 indicates that there is no CAM modification on C residue C(5) and a possible dehydration on the S1 residue. This pattern would allow for the presence of ring A, but not ring B and C. But note that those are 2 out of only 6 PSMs for this peptide and that there are 166 PSMs for peptide SACTPGCPTGILMTCPLK ruling out the presence of ring A.

Supplementary Tables S1-S3
Lists of PSMs for modified NsoA1-3 core peptides generated by missed cleavages of trypsin

Table S1:
YK<u>SKSAC</u>TPGCPTGILMTCPLKTATCGCHITGK peptide
(3 missed cleavages, potential ring A underlined)

	#dehydrations			
**CAM**	**1**	**6**	**7**	**8**
**0**		1	17	3
**1**		2		
**4**	1			

Table S2:
<u>SKSAC</u>TPGCPTGILMTCPLKTATCGCHITGK peptide
(2 missed cleavages)

	#dehydrations			
**CAM**	**5**	**6**	**7**	**8**
**0**		7	2	1
**1**		2		
**3**	1			

Table S3:
<u>SAC</u>TPGCPTGILMTCPLKTATCGCHITGK peptide
(1 missed cleavage, cleaved inside potential ring A)

	#dehydrations					
**CAM**	**1**	**2**	**3**	**4**	**5**	**6**
**1**				1	5	9
**2**			9	4	4	1
**3**			5	2	1	
**4**	9	11	5			1
**5**				1		

Table S4-S9: Fragmentation tables for spectra shown in Figures 3 and 5 (main text) All tables have been exported from Scaffold file 3, also provided as Supplementary Info

Fragmentation Table S4 for spectrum in panel a) in **Fig. 3**
Peptide sequence YKSKSACTPGCPTGILMTCPLKTATCGCHITGK (no CAM, 8 dehydrations)

B	B Ions	B+2H	B-NH3	B-H2O	AA	Y Ions	Y+2H	Y-NH3	Y-H2O	Y
1	164.1	82.5		146.1	Y	3,227.5	1,614.3	3,210.5	3,209.5	33
2	292.2	146.6	275.1	274.2	K	3,064.5	1,532.7	3,047.4	3,046.5	32
3	361.2	181.1	344.2	343.2	S-18	2,936.4	1,468.7	2,919.3	2,918.4	31
4	489.3	245.1	472.3	471.3	K	2,867.3	1,434.2	2,850.3	2,849.3	30
5	558.3	279.7	541.3	540.3	S-18	2,739.3	1,370.1	2,722.2	2,721.2	29
6	629.3	315.2	612.3	611.3	A	2,670.2	1,335.6	2,653.2	2,652.2	28
7	732.3	366.7	715.3	714.3	C	2,599.2	1,300.1	2,582.2	2,581.2	27
8	815.4	408.2	798.4	797.4	T-18	2,496.2	1,248.6	2,479.2	2,478.2	26
9	912.4	456.7	895.4	894.4	P	2,413.1	1,207.1	2,396.1	2,395.1	25
10	969.5	485.2	952.4	951.5	G	2,316.1	1,158.6	2,299.1	2,298.1	24
11	1,072.5	536.7	1,055.4	1,054.5	C	2,259.1	1,130.0	2,242.0	2,241.1	23
12	1,169.5	585.3	1,152.5	1,151.5	P	2,156.1	1,078.5	2,139.0	2,138.1	22
13	1,252.6	626.8	1,235.5	1,234.5	T-18	2,059.0	1,030.0	2,042.0	2,041.0	21
14	1,309.6	655.3	1,292.6	1,291.6	G	1,976.0	988.5	1,958.9	1,958.0	20
15	1,422.7	711.8	1,405.6	1,404.7	I	1,919.0	960.0	1,901.9	1,900.9	19
16	1,535.8	768.4	1,518.7	1,517.7	L	1,805.9	903.4	1,788.8	1,787.9	18
17	1,666.8	833.9	1,649.8	1,648.8	M	1,692.8	846.9	1,675.8	1,674.8	17
18	1,749.8	875.4	1,732.8	1,731.8	T-18	1,561.7	781.4	1,544.7	1,543.7	16
19	1,852.8	926.9	1,835.8	1,834.8	C	1,478.7	739.9	1,461.7	1,460.7	15
20	1,949.9	975.4	1,932.9	1,931.9	P	1,375.7	688.4	1,358.7	1,357.7	14
21	2,063.0	1,032.0	2,045.9	2,045.0	L	1,278.6	639.8	1,261.6	1,260.6	13
22	2,191.1	1,096.0	2,174.0	2,173.1	K	1,165.6	583.3	1,148.5	1,147.6	12
23	2,274.1	1,137.6	2,257.1	2,256.1	T-18	1,037.5	519.2	1,020.4	1,019.5	11
24	2,345.1	1,173.1	2,328.1	2,327.1	A	954.4	477.7	937.4	936.4	10
25	2,428.2	1,214.6	2,411.2	2,410.2	T-18	883.4	442.2	866.4	865.4	9
26	2,531.2	1,266.1	2,514.2	2,513.2	C	800.4	400.7	783.3	782.3	8
27	2,588.2	1,294.6	2,571.2	2,570.2	G	697.3	349.2	680.3	679.3	7
28	2,691.2	1,346.1	2,674.2	2,673.2	C	640.3	320.7	623.3	622.3	6
29	2,828.3	1,414.6	2,811.3	2,810.3	H	537.3	269.2	520.3	519.3	5
30	2,941.4	1,471.2	2,924.3	2,923.4	I	400.3	200.6	383.2	382.2	4
31	3,024.4	1,512.7	3,007.4	3,006.4	T-18	287.2	144.1	270.1	269.2	3
32	3,081.4	1,541.2	3,064.4	3,063.4	G	204.1	102.6	187.1	186.1	2
33	3,227.5	1,614.3	3,210.5	3,209.5	K	147.1	74.1	130.1	129.1	1

Fragmentation Table S5 for spectrum in panel b) in Fig. 3
Peptide sequence YKSKSACTPGCPTGILMTCPLKTATCGCHITGK (no CAM, 7 dehydrations)

B	B Ions	B+2H	B-NH3	B-H2O	AA	Y Ions	Y+2H	Y-NH3	Y-H2O	Y
1	164.1	82.5		146.1	Y	3,245.5	1,623.3	3,228.5	3,227.5	33
2	292.2	146.6	275.1	274.2	K	3,082.5	1,541.7	3,065.4	3,064.5	32
3	361.2	181.1	344.2	343.2	S-18	2,954.4	1,477.7	2,937.4	2,936.4	31
4	489.3	245.1	472.3	471.3	K	2,885.4	1,443.2	2,868.3	2,867.3	30
5	576.3	288.7	559.3	558.3	S	2,757.3	1,379.1	2,740.2	2,739.3	29
6	647.4	324.2	630.3	629.3	A	2,670.2	1,335.6	2,653.2	2,652.2	28
7	750.4	375.7	733.3	732.3	C	2,599.2	1,300.1	2,582.2	2,581.2	27
8	833.4	417.2	816.4	815.4	T-18	2,496.2	1,248.6	2,479.2	2,478.2	26
9	930.5	465.7	913.4	912.4	P	2,413.1	1,207.1	2,396.1	2,395.1	25
10	987.5	494.2	970.4	969.5	G	2,316.1	1,158.6	2,299.1	2,298.1	24
11	1,090.5	545.7	1,073.5	1,072.5	C	2,259.1	1,130.0	2,242.0	2,241.1	23
12	1,187.5	594.3	1,170.5	1,169.5	P	2,156.1	1,078.5	2,139.0	2,138.1	22
13	1,270.6	635.8	1,253.5	1,252.6	T-18	2,059.0	1,030.0	2,042.0	2,041.0	21
14	1,327.6	664.3	1,310.6	1,309.6	G	1,976.0	988.5	1,958.9	1,958.0	20
15	1,440.7	720.8	1,423.6	1,422.7	I	1,919.0	960.0	1,901.9	1,900.9	19
16	1,553.8	777.4	1,536.7	1,535.8	L	1,805.9	903.4	1,788.8	1,787.9	18
17	1,684.8	842.9	1,667.8	1,666.8	M	1,692.8	846.9	1,675.8	1,674.8	17
18	1,767.8	884.4	1,750.8	1,749.8	T-18	1,561.7	781.4	1,544.7	1,543.7	16
19	1,870.8	935.9	1,853.8	1,852.8	C	1,478.7	739.9	1,461.7	1,460.7	15
20	1,967.9	984.5	1,950.9	1,949.9	P	1,375.7	688.4	1,358.7	1,357.7	14
21	2,081.0	1,041.0	2,064.0	2,063.0	L	1,278.6	639.8	1,261.6	1,260.6	13
22	2,209.1	1,105.0	2,192.1	2,191.1	K	1,165.6	583.3	1,148.5	1,147.6	12
23	2,292.1	1,146.6	2,275.1	2,274.1	T-18	1,037.5	519.2	1,020.4	1,019.5	11
24	2,363.2	1,182.1	2,346.1	2,345.1	A	954.4	477.7	937.4	936.4	10
25	2,446.2	1,223.6	2,429.2	2,428.2	T-18	883.4	442.2	866.4	865.4	9
26	2,549.2	1,275.1	2,532.2	2,531.2	C	800.4	400.7	783.3	782.3	8
27	2,606.2	1,303.6	2,589.2	2,588.2	G	697.3	349.2	680.3	679.3	7
28	2,709.2	1,355.1	2,692.2	2,691.2	C	640.3	320.7	623.3	622.3	6
29	2,846.3	1,423.6	2,829.3	2,828.3	H	537.3	269.2	520.3	519.3	5
30	2,959.4	1,480.2	2,942.3	2,941.4	I	400.3	200.6	383.2	382.2	4
31	3,042.4	1,521.7	3,025.4	3,024.4	T-18	287.2	144.1	270.1	269.2	3
32	3,099.4	1,550.2	3,082.4	3,081.4	G	204.1	102.6	187.1	186.1	2
33	3,245.5	1,623.3	3,228.5	3,227.5	K	147.1	74.1	130.1	129.1	1

Fragmentation Table S6 for spectrum in panel c) in **Fig. 3**

Peptide sequence SKSACTPGCPTGILMTCPLKTATCGCHITGK (no CAM, 6 dehydrations)

B	B Ions	B+2H	B-NH3	B-H2O	AA	Y Ions	Y+2H	Y-NH3	Y-H2O	Y
1	70.0	35.5		52.0	S-18	2,972.4	1,486.7	2,955.4	2,954.4	31
2	198.1	99.6	181.1	180.1	K	2,903.4	1,452.2	2,886.3	2,885.4	30
3	285.2	143.1	268.1	267.1	S	2,775.3	1,388.1	2,758.2	2,757.3	29
4	356.2	178.6	339.2	338.2	A	2,688.2	1,344.6	2,671.2	2,670.2	28
5	459.2	230.1	442.2	441.2	C	2,617.2	1,309.1	2,600.2	2,599.2	27
6	560.2	280.6	543.2	542.2	T	2,514.2	1,257.6	2,497.2	2,496.2	26
7	657.3	329.2	640.3	639.3	P	2,413.1	1,207.1	2,396.1	2,395.1	25
8	714.3	357.7	697.3	696.3	G	2,316.1	1,158.6	2,299.1	2,298.1	24
9	817.3	409.2	800.3	799.3	C	2,259.1	1,130.0	2,242.0	2,241.1	23
10	914.4	457.7	897.4	896.4	P	2,156.1	1,078.5	2,139.0	2,138.1	22
11	997.4	499.2	980.4	979.4	T-18	2,059.0	1,030.0	2,042.0	2,041.0	21
12	1,054.4	527.7	1,037.4	1,036.4	G	1,976.0	988.5	1,958.9	1,958.0	20
13	1,167.5	584.3	1,150.5	1,149.5	I	1,919.0	960.0	1,901.9	1,900.9	19
14	1,280.6	640.8	1,263.6	1,262.6	L	1,805.9	903.4	1,788.8	1,787.9	18
15	1,411.7	706.3	1,394.6	1,393.6	M	1,692.8	846.9	1,675.8	1,674.8	17
16	1,494.7	747.8	1,477.7	1,476.7	T-18	1,561.7	781.4	1,544.7	1,543.7	16
17	1,597.7	799.4	1,580.7	1,579.7	C	1,478.7	739.9	1,461.7	1,460.7	15
18	1,694.8	847.9	1,677.7	1,676.7	P	1,375.7	688.4	1,358.7	1,357.7	14
19	1,807.8	904.4	1,790.8	1,789.8	L	1,278.6	639.8	1,261.6	1,260.6	13
20	1,935.9	968.5	1,918.9	1,917.9	K	1,165.6	583.3	1,148.5	1,147.6	12
21	2,019.0	1,010.0	2,001.9	2,001.0	T-18	1,037.5	519.2	1,020.4	1,019.5	11
22	2,090.0	1,045.5	2,073.0	2,072.0	A	954.4	477.7	937.4	936.4	10
23	2,173.0	1,087.0	2,156.0	2,155.0	T-18	883.4	442.2	866.4	865.4	9
24	2,276.1	1,138.5	2,259.0	2,258.0	C	800.4	400.7	783.3	782.3	8
25	2,333.1	1,167.0	2,316.0	2,315.1	G	697.3	349.2	680.3	679.3	7
26	2,436.1	1,218.5	2,419.1	2,418.1	C	640.3	320.7	623.3	622.3	6
27	2,573.1	1,287.1	2,556.1	2,555.1	H	537.3	269.2	520.3	519.3	5
28	2,686.2	1,343.6	2,669.2	2,668.2	I	400.3	200.6	383.2	382.2	4
29	2,769.3	1,385.1	2,752.2	2,751.3	T-18	287.2	144.1	270.1	269.2	3
30	2,826.3	1,413.6	2,809.3	2,808.3	G	204.1	102.6	187.1		2
31	2,972.4	1,486.7	2,955.4	2,954.4	K	147.1	74.1	130.1		1

Fragmentation Table S7 for spectrum in panel d) in **Fig. 3**
Peptide sequence SACTPGCPTGILMTCPLKTATCGCHITGK (3 CAM, no dehydration)

B	B Ions	B+2H	B-NH3	B-H2O	AA	Y Ions	Y+2H	Y-NH3	Y-H2O	Y
1	88.0	44.5		70.0	S	2,982.4	1,491.7	2,965.3	2,964.3	29
2	159.1	80.0		141.1	A	2,895.3	1,448.2	2,878.3	2,877.3	28
3	319.1	160.1		301.1	C+57	2,824.3	1,412.6	2,807.3	2,806.3	27
4	420.2	210.6		402.1	T	2,664.3	1,332.6	2,647.2	2,646.2	26
5	517.2	259.1		499.2	P	2,563.2	1,282.1	2,546.2	2,545.2	25
6	574.2	287.6		556.2	G	2,466.2	1,233.6	2,449.1	2,448.1	24
7	734.3	367.6		716.2	C+57	2,409.1	1,205.1	2,392.1	2,391.1	23
8	831.3	416.2		813.3	P	2,249.1	1,125.1	2,232.1	2,231.1	22
9	932.4	466.7		914.3	T	2,152.1	1,076.5	2,135.0	2,134.0	21
10	989.4	495.2		971.4	G	2,051.0	1,026.0	2,034.0	2,033.0	20
11	1,102.5	551.7		1,084.5	I	1,994.0	997.5	1,977.0	1,976.0	19
12	1,215.5	608.3		1,197.5	L	1,880.9	941.0	1,863.9	1,862.9	18
13	1,346.6	673.8		1,328.6	M	1,767.8	884.4	1,750.8	1,749.8	17
14	1,429.6	715.3		1,411.6	T-18	1,636.8	818.9	1,619.7	1,618.8	16
15	1,589.7	795.3		1,571.6	C+57	1,553.7	777.4	1,536.7	1,535.7	15
16	1,686.7	843.9		1,668.7	P	1,393.7	697.4	1,376.7	1,375.7	14
17	1,799.8	900.4		1,781.8	L	1,296.7	648.8	1,279.6	1,278.6	13
18	1,927.9	964.4	1,910.9	1,909.9	K	1,183.6	592.3	1,166.5	1,165.6	12
19	2,010.9	1,006.0	1,993.9	1,992.9	T-18	1,055.5	528.2	1,038.4	1,037.5	11
20	2,082.0	1,041.5	2,064.9	2,064.0	A	972.4	486.7	955.4	954.4	10
21	2,165.0	1,083.0	2,148.0	2,147.0	T-18	901.4	451.2	884.4	883.4	9
22	2,268.0	1,134.5	2,251.0	2,250.0	C	818.4	409.7	801.3	800.4	8
23	2,325.0	1,163.0	2,308.0	2,307.0	G	715.4	358.2	698.3	697.3	7
24	2,428.0	1,214.5	2,411.0	2,410.0	C	658.3	329.7	641.3	640.3	6
25	2,565.1	1,283.1	2,548.1	2,547.1	H	555.3	278.2	538.3	537.3	5
26	2,678.2	1,339.6	2,661.2	2,660.2	I	418.3	209.6	401.2	400.3	4
27	2,779.2	1,390.1	2,762.2	2,761.2	T	305.2	153.1	288.2	287.2	3
28	2,836.3	1,418.6	2,819.2	2,818.2	G	204.1	102.6	187.1		2
29	2,982.4	1,491.7	2,965.3	2,964.3	K	147.1	74.1	130.1		1

Fragmentation Table S8 for spectrum in panel a) in **Fig. 5**
Peptide sequence TATCGCHITGK (3 dehydrations)

B	B Ions	B+2H	B-NH3	B-H2O	AA	Y Ions	Y+2H	Y-NH3	Y-H2O	Y
1	84.0			66.0	T-18	1,037.5	519.2	1,020.4	1,019.5	11
2	155.1			137.1	A	954.4	477.7	937.4	936.4	10
3	238.1			220.1	T-18	883.4	442.2	866.4	865.4	9
4	341.1			323.1	C	800.4	400.7	783.3	782.3	8
5	398.1			380.1	G	697.3	349.2	680.3	679.3	7
6	501.2	251.1		483.1	C	640.3	320.7	623.3	622.3	6
7	638.2	319.6		620.2	H	537.3	269.2	520.3	519.3	5
8	751.3	376.2		733.3	I	400.3		383.2	382.2	4
9	834.3	417.7		816.3	T-18	287.2		270.1	269.2	3
10	891.4	446.2		873.3	G	204.1		187.1		2
11	1,037.5	519.2	1,020.4	1,019.5	K	147.1		130.1		1

Fragmentation Table S9 for spectrum in panel b) in **Fig. 5**
Peptide sequence TATCGCHITGK (2 dehydrations)

B	B Ions	B+2H	B-NH3	B-H2O	AA	Y Ions	Y+2H	Y-NH3	Y-H2O	Y
1	84.0			66.0	T-18	1,055.5	528.2	1,038.4	1,037.5	11
2	155.1			137.1	A	972.4	486.7	955.4	954.4	10
3	238.1			220.1	T-18	901.4	451.2	884.4	883.4	9
4	341.1			323.1	C	818.4	409.7	801.3	800.4	8
5	398.1			380.1	G	715.4	358.2	698.3	697.3	7
6	501.2	251.1		483.1	C	658.3	329.7	641.3	640.3	6
7	638.2	319.6		620.2	H	555.3	278.2	538.3	537.3	5
8	751.3	376.2		733.3	I	418.3		401.2	400.3	4
9	852.3	426.7		834.3	T	305.2		288.2	287.2	3
10	909.4	455.2		891.4	G	204.1		187.1		2
11	1,055.5	528.2	1,038.4	1,037.5	K	147.1		130.1		1
